# USP37 regulates DNA damage response through stabilizing and deubiquitinating BLM

**DOI:** 10.1093/nar/gkab842

**Published:** 2021-10-04

**Authors:** Chenming Wu, Yiming Chang, Junliang Chen, Yang Su, Lei Li, Yuping Chen, Yunhui Li, Jinhuan Wu, Jinzhou Huang, Fei Zhao, Wenrui Wang, Hui Yin, Shunli Wang, Mingpeng Jin, Zhenkun Lou, Wei-Guo Zhu, Kuntian Luo, Jie Zhang, Jian Yuan

**Affiliations:** Key Laboratory of Arrhythmias of the Ministry of Education of China, Research Center for Translational Medicine, East Hospital, Tongji University School of Medicine, Shanghai 200120, China; Department of Biochemistry and Molecular Biology, Tongji University School of Medicine, Shanghai 200120, China; Jinzhou Medical University, Jinzhou 121001, China; MOE Laboratory of Biosystems Homeostasis and Protection and Innovation Center for Cell Signaling Network, Life Sciences Institute, Zhejiang University, Hangzhou 310058, China; Key Laboratory of Arrhythmias of the Ministry of Education of China, Research Center for Translational Medicine, East Hospital, Tongji University School of Medicine, Shanghai 200120, China; Key Laboratory of Arrhythmias of the Ministry of Education of China, Research Center for Translational Medicine, East Hospital, Tongji University School of Medicine, Shanghai 200120, China; Key Laboratory of Arrhythmias of the Ministry of Education of China, Research Center for Translational Medicine, East Hospital, Tongji University School of Medicine, Shanghai 200120, China; Key Laboratory of Arrhythmias of the Ministry of Education of China, Research Center for Translational Medicine, East Hospital, Tongji University School of Medicine, Shanghai 200120, China; Key Laboratory of Arrhythmias of the Ministry of Education of China, Research Center for Translational Medicine, East Hospital, Tongji University School of Medicine, Shanghai 200120, China; Department of Oncology, Mayo Clinic, Rochester, MN 55905, USA; Department of Oncology, Mayo Clinic, Rochester, MN 55905, USA; Department of Biotechnology, Bengbu Medical College, Anhui 233030, China; Department of Thoracic Surgery, The First Affiliated Hospital of Nanchang University, Nanchang 330006, China; Department of Pathology,Shanghai East Hospital, Tongji University, Shanghai 200120, China; Key Laboratory of Arrhythmias of the Ministry of Education of China, Research Center for Translational Medicine, East Hospital, Tongji University School of Medicine, Shanghai 200120, China; Department of Oncology, Mayo Clinic, Rochester, MN 55905, USA; Guangdong Key Laboratory of Genome Instability and Human Disease, Shenzhen University Carson Cancer Center, Department of Biochemistry and Molecular Biology, Shenzhen University School of Medicine, Shenzhen 518060, China; Department of Oncology, Mayo Clinic, Rochester, MN 55905, USA; Department of Thoracic Surgery, Shanghai Chest Hospital,Shanghai Jiao Tong University, Shanghai, China; Key Laboratory of Arrhythmias of the Ministry of Education of China, Research Center for Translational Medicine, East Hospital, Tongji University School of Medicine, Shanghai 200120, China; Department of Biochemistry and Molecular Biology, Tongji University School of Medicine, Shanghai 200120, China

## Abstract

The human RecQ helicase BLM is involved in the DNA damage response, DNA metabolism, and genetic stability. Loss of function mutations in BLM cause the genetic instability/cancer predisposition syndrome Bloom syndrome. However, the molecular mechanism underlying the regulation of BLM in cancers remains largely elusive. Here, we demonstrate that the deubiquitinating enzyme USP37 interacts with BLM and that USP37 deubiquitinates and stabilizes BLM, thereby sustaining the DNA damage response (DDR). Mechanistically, DNA double-strand breaks (DSB) promotes ATM phosphorylation of USP37 and enhances the binding between USP37 and BLM. Moreover, knockdown of USP37 increases BLM polyubiquitination, accelerates its proteolysis, and impairs its function in DNA damage response. This leads to enhanced DNA damage and sensitizes breast cancer cells to DNA-damaging agents in both cell culture and *in vivo* mouse models. Collectively, our results establish a novel molecular mechanism for the USP37–BLM axis in regulating DSB repair with an important role in chemotherapy and radiotherapy response in human cancers.

## INTRODUCTION

Maintaining genome stability is essential for the health of organisms (1,2). The genome is constantly under attack from intrinsic and extrinsic DNA damaging agents such as reactive radicals, radiation and carcinogens (3,4). To ensure genomic stability, eukaryotic cells have developed a sophisticated molecular machinery, called the DNA damage response (DDR), to detect, signal and repair the DNA lesions (1,3,5,6). The DDR executes a variety of responses such as cell-cycle checkpoint activation, DNA repair, apoptosis and senescence (5–8). In cells, DNA double-strand breaks (DSBs) have been considered to be the most cytotoxic type of DNA damage. They are responsible for the development of chromosomal aberrations or mutations and contribute to the development of diseases such as cancer because of inappropriate or deficient repair of DSBs (9–11). Therefore, defects or dysfunction in the DDR leads to genomic instability and tumorigenesis. However, DDR defects also confer increased sensitivity to DNA-damaging cancer therapy (10,12,13).

RecQ helicases are a highly conserved family of DNA processing enzymes. There are five RecQ helicases including RecQ1, BLM, WRN, RecQ4 and RecQ5 in humans (14). These members are involved in DNA metabolism, genetic stability and the DNA damage response, and play critical roles in the maintenance of genome stability (15–18). The mutation or loss of three genes BLM, WRN or RecQ4 leads to related diseases, which are Bloom syndrome (BS), Werner syndrome (WS) or Rothmund-Thomson syndrome (RTS), respectively (19–21). Patients with these diseases have profound developmental abnormalities and an increased susceptibility to cancer (22,23). BS patients are prone to develop multiple malignancies including breast, prostate and lung cancers (24). The defective protein in BS, BLM, is an upstream sensor protein in the DNA damage signaling cascade (25), plays important roles in the regulation of key DNA metabolism processes like DNA replication, recombination and repair (15). Increasing evidence suggests that BLM is involved in DSB repair; in accordance with this, the loss function of BLM in cancer cells causes hypersensitivity to DNA-damaging agents that directly or indirectly generate DSBs (26–29). BLM has been shown to be ubiquitinated, which in turn induces defect in DDR pathway (30,31). However, the deubiquitination process which regulates BLM in the DDR is still not clear.

In this study, we found that ubiquitin specific peptidase 37 (USP37) functions as the deubiquitinase of BLM and regulates the DDR. Mechanistically, USP37 is phosphorylated by ATM following DNA damage, which increases the binding between USP37 and BLM and leads to BLM deubiquitylation. Knockdown of USP37 impairs the DDR through BLM and results in increased sensitivity to cisplatin or IR treatment in breast cancer cells. In addition, we found that USP37 expression is up-regulated in breast cancer tissue arrays. In addition, GEO database showed that high expression of USP37 is significantly correlated with poor survival in breast cancer patients. Furthermore, USP37 knockdown sensitizes cancer cells to DNA-damaging agents in xenograft models, suggesting that the USP37–BLM axis may provide new therapeutic targets for overcoming chemo or radiotherapy resistance in breast cancer.

## MATERIALS AND METHODS

### Cell culture, plasmids and antibodies

HEK293T, GP293, U-2OS, BT-549, T47D, MDA-MB-436, MDA-MB-468, MDA-MB-231, MCF7, Hela, ER-AsiSI U2OS and MCF10A cell lines were cultured in DMEM, RPMI-1640, McCoy's 5A or Ham's F-12 supplemented with 10% FBS, 100 U of penicillin, and 100 μg/ml streptomycin. These cells were originally from ATCC.

Wild-type HA-FLAG-USP37 plasmid was purchased from Addgene (plasmid #22602), generated by Dr Wade Harper from Harvard University. HA-FLAG-USP37 and MYC-BLM were subcloned into 3*FLAG-Plvx3 vector. All site mutants were constructed using the site-directed mutagenesis Kit (Stratagene) and verifified by DNA sequencing. MYC-tagged BLM was a gift from Junjie Chen in the University of Texas MD Anderson Cancer Center. The sequences of shRNAs from Sigma are listed below: USP37 shRNA#1: CCGGATTTGCAGAAGATGATA, USP37 shRNA#2: CCCTAACTTCTCTGGCCTATT, BLM shRNA: GCCTTTATTCAATACCCATTT. RAP80 shRNA: TGAGAAGGAAGTAGCTATTTC. The siRNAs sequences are as follows: USP37 siRNA#1: CAAAAGAGCUACCGAGUUA. USP37 siRNA#2: GCAUACACUUGCCCUGUUA. BLM siRNA: CAGGAUGGCUGUCAGGUUA.

Anti-USP37 antibodies were purchased from Proteintech (18465-1-AP) and Bethyl Laboratories (A300-927A). Anti-RAP80 (A303-763A) and anti-Phospho RPA32 (A300-245A) antibodies from Bethyl Laboratories. Anti-BLM antibodies were obtained from Abcam (ab476,ab2179) and Santa Cruz Biotechnology (sc-376237). Anti-Myc (9E10) antibody was purchased from Covance. Anti-HA (H3663), anti-Flag (m2), and anti-β-actin (A1978) antibodies were obtained from Sigma. Anti-His (sc-8036), anti-Ubiquitin (sc-8017), anti-RPA32 (sc-56770) and anti- cyclin A (sc-239) antibodies were purchased from Santa Cruz Biotechnology. Anti-pT68-CHK2 (#2661), anti-CHK2 (#3440), anti–γH2AX (#9718) and anti-pS/TQ-ATM/ATR (#6966s) antibodies were obtained from Cell Signaling Technology. Anti-Histone H3(ab1791) and anti-ATM(ab32420) antibodies were purchased from Abcam. Anti-γH2AX antibody(05-636) was obtained from Millipore. Alexa Fluor^®^ 488-AffiniPure Goat Anti-Mouse IgG (H + L) (115-545-062), Alexa Fluor® 488-AffiniPure Goat Anti-Rabbit IgG (H + L) (111-545-045), Rhodamine Red-X-AffiniPure Goat Anti-Mouse IgG (H + L) (115-295-146) and Rhodamine Red-X-AffiniPure Goat Anti-Rabbit IgG (H + L) (111-295-144) were purchased from Jackson Lab.

### Viral infections

Packaging of lentivirus and retrovirus as well as subsequent infection of various cells were performed according to our previously described experimental procedures (32).

### Protein stability assays

For protein turnover analysis in cells, a CHX (cycloheximide)-based assays were carried out according to our previously described experimental procedures (32).

### Deubiquitination assays *in vivo* and *in vitro*

The *in vivo* and *in vitro* deubiquitination assays were performed as described previously (32). Briefly, for the *in vivo* deubiquitination assays, we transfected wild type (WT)-USP37, CA mutant-USP37 and Myc-ub in HEK293T cells. After transfection 48h later, cells were treated with MG132 for 4 h before being harvested and the cells were lysed in the SDS buffer and boiled. Once diluted (1:10 ratio) with NETN buffer containing 1 mM iodoacetamide and 20 mM NEM, the lysates were immunoprecipitated with the indicated antibodies for 8 h (4°C). After NETN buffer washing, the immunocomplexes were subjected to western blot.

For the *in vitro* assays, we purified His-Ub conjugated Myc-BLM from HEK293T cells through multiple steps as described previously (32). The purified USP37 WT and CA mutant were incubated with Ub-Myc-BLM in deubiquitination buffer for 30 min (30°C).

### Cell survival assays

For cell survival assays, infected cells (5 × 10^3^ cells/well) per well were plated in 96-well plates and treated with cisplatin or IR. After incubated with drugs for 48h, the survival cells were detected by Cell Counting Kit-8 (CCK8) assay according to the manufacturer's instructions (Dojindo, Janpan).

### Colony formation assays

Colony formation assays were performed as described previously (32). Breifly, cells were treated with cisplatin at indicated concentrations for 8 h or were exposed to IR, and then washed with PBS,changed fresh DMEM or McCoy's 5A overnight. The following day plated in six-well plate (800 cells per well) cultured for 9–12 days at 37°C. We washed the visible colonies with PBS, fixed with methanol for 30 min, stained with 5% GIEMSA and counted.

### Animal experiment

The nude mice were maintained in specific pathogen-free environments. USP37 knockdown MDA-MB-231 cells stably expressing USP37 WT, S114A or EV were subcutaneously injected into the 6-week-old female BALB/C nude mice using 18-gauge needles. Each mouse received two injections of 200 μl mixture (3 × 10^6^ cells in 140 μl of 1× PBS and 60 μl of Matrigel). When the mean size of tumor volume in each randomized group reached ∼100 mm^3^, the mice were randomly treated with cisplatin (20 mg/kg) every other day. Tumor volumes were measured every 7 days using calipers, and tumor volumes were calculated using the formula length × (width)^2^. Mice were sacrificed for tumor dissection on day 28 of treatment. All experimental procedures were performed under the approval of the Medical Ethics Committee of Shanghai East Hospital (Shanghai, China).

### IHC assays

The tissue microarray of breast cancer samples were purchased from Alenabio (www.Alenabio.com) (BRN801b and BC081120e) and the detailed information including stage, age, and classification and so on in the Supplementary Table S1. Immunohistochemical (IHC) staining of USP37 (Proteintech, 18465-1-AP, dilution 1:100) and BLM (Santa Cruz Biotechnology,c-376237,dilution 1:50) was carried out according to the standard protocol, as described previously (32). The immunostaining was blindly scored by pathologists. The IHC score was judged as described in our previous publication. *χ^2^* test and the Pearson correlation coefficient were used for statistical analysis of the correlation between USP37 and BLM.

### Cell cycle analysis

Cells were harvested and fixed in 70% ice-cold ethanol over night, and treated with RNase A, stained with propidium iodide and analyzed on an Beckman CytoFLEX B4-R3-V3 and data analyzed with FlowJo software.

### *In vitro* kinase assay

In vitro kinase assays were performed as described previously (33). Breifly, purified WT-USP37, S114A or S114E mutant was incubated with ATM at 30°C in buffers containing [25 mM Tris–HCl (pH 7.4), 2 mM adenosine 5′-triphosphate (ATP), 5 mM MgCl_2_, 5 mM MnCl_2_ and 0.1 mM dithiothreitol (DTT)] for *in vitro* kinase assay,

### DNA resection assays

The percentage of single-stranded DNA (ssDNA%) generated by resection was measured as previously described (34,35). Briefly, ER-AsiSI U2OS cells were treated with 5 μM 4-OHT for 4 h, and then genomic DNA (gDNA) was extracted with genomic DNA Extraction Kit (Tiangen Biotech,Beijing China). After that, 500 ng genomic DNA sample was digested or mock digested with 20 units of restriction enzymes BsrGI (New England Biolabs) at 37°C overnight. 1 μl DNA were used as templates in 10 μl of qPCR reaction containing 5 μl of 2× PerfectStart II Probe qPCR SupperMix (TransGen Biotech,Beijing, China), 0.2 μM of each primer and 0.2 μM probe. The sequences of qPCR primers and probes are shown in Supplementary Table S2. The ssDNA% generated by resection at selected sites was calculated with the following equation: ssDNA% = 1/(2^(△Ct –1) + 0.5) × 100.

### Immunofluorescence staining

U-2OS cells were seeded on coverslips for 24 h before experiments. Cells were permeabilized for 10min at room temperature with 0.1% Triton X-100 and then fixed by 3% paraformaldehyde for 10min. And then, coverslips were washed twice with PBS. Following this, cells were blocked with 5% goat serum for 30 min and then washed one time with PBS. And incubated with primary antibodies, diluted in 3% BSA in PBS, at 4°C overnight. The following day, after washing with PBS for three times, secondary antibodies diluted in 3% BSA in PBS for 1 h at room temperature.

Finally, the coverslips were washed three times with PBS and then mounted with Prolong Diamond antifade mountant (Thermo Fisher). The foci intensity was quantified with ImageJ software.

### Ultra-fine anaphase bridges (UFBs) detection

The detection of UFBs was performed as described previously (36). Briefly, U-2OS cells stably expressing the indicated shRNAs were treated with cisplatin (3 μM for 1 h), and released into fresh media for 24 h. Cells were then fixed with PTEMF buffer (20 mM PIPES pH 6.8, 0.2% Triton X-100, 1 mM MgCl_2_, 10 mM EGTA and 4% paraformaldehyde) for 15min, and then permeabilized with 0.2% Triton X-100 in PBS for 5 min. Next, cells were blocked with 3% BSA dissolve in PBS for 30 min. Following this, cells were incubated with BLM antibody (1:100; Abcam ab2179), diluted in 3% BSA in PBS, at 4°C overnight. The following day, after washing with PBS, secondary antibodies diluted in 3% BSA in PBS for 1 h at room temperature. The coverslips were washed three times with PBS and then mounted with Prolong Diamond antifade mountant (Thermo Fisher).

### Sister chromatid exchange assay

Sister chromatid exchange assays (SCE) were performed as previously described (37,38). Briefly,Hela cells were treated with cisplatin (10 μM) for 1 h and then exposed to 100 μM BrdU for 40 h. During the BrdU treatment,colcemid (Sigma) was added(0.2 μg/ml) to accumulate mitotic cells 2 h before harvesting cells and then harvested the metaphase cells by mitotic shake off. Cells were then swollen in pre-heat (37°C) hypotonic solution (46.5 mM KCl, 8.5 mM Na.citrate) for 15 min, fixed with 3:1 (vol/vol) methanol/acetic acid. Following this,fixed cell suspension was dropped onto a glass slide and air-dried for 2 days. After that, slides were heated at 88°C for 10 min in buffer [1.0 M NaH_2_PO_4_ (pH 8.0)], rinsed in distilled water, and stained with 5% Giemsa for 8 min. Finally,slides were then rinsed and air-dried.

### Statistics

All data are based on experiments performed at least in triplicate for cell survival and data are presented as the mean ± SD (*n* = 6). Statistical significance was assessed by two-way ANOVA with Tukey's multiple comparisons test in cell survival assays.

For the animal study, data are presented as the mean ± SEM of 6 mice. Statistical analyses were performed with the χ2 test or the Student's t-test (two-tailed unpaired). In the figures, statistical significance is presented by: n.s.: not significant,*P < 0.05; **P < 0.01, ***P < 0.001, ****P < 0.0001.

## RESULTS

### USP37 interacts with BLM

**Figure 1.**
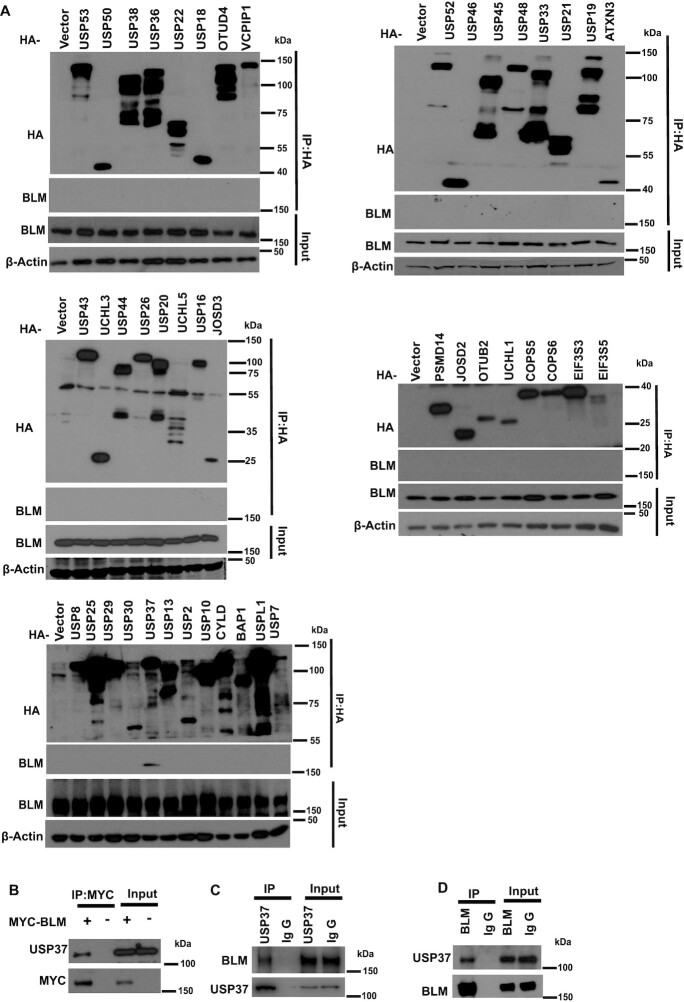
USP37 interacts with BLM. (**A**) 293T cells were transfected with HA-tagged cDNA as indicated and then treated with 10 μM MG132 for 4 h before performing Co-immunoprecipitation (Co-IP) assays. Immunoblotted with the indicated antibodies. (**B**) Interaction between transfected Myc-tagged-BLM and endogenous USP37. Lysates from HEK293T cells expressing Myc-BLM were subjected to co-IP assays using the USP37 antiboddy and Myc antibody followed by western blot analysis. (C, D) Interaction between endogenous USP37 and BLM. HEK293T cell were collected and subjected to immunoprecipitation using control IgG, (**C**) USP37 antibody or (**D**) BLM antibody. Blots were probed with the indicated antibodies.

In order to identify the potential deubiquitinase(s) that regulate(s) BLM, we transfected HEK293T cells with a panel of HA-tagged deubiquitinases individually and immunoprecipitated with HA antibody-conjugated beads. We found that only HA-tagged USP37 bound with endogenous BLM (Figure 1A). Furthermore, the reciprocal immunoprecipitation of MYC-tagged BLM in HEK293T cells pulled down USP37 (Figure 1B). In addition, we examined the endogenous interaction between USP37 and BLM in HEK293T cells and confirmed that USP37 interacted with BLM (Figure 1C, D). These data suggest that USP37 associates with BLM.

### USP37 promotes BLM stabilization

**Figure 2.**
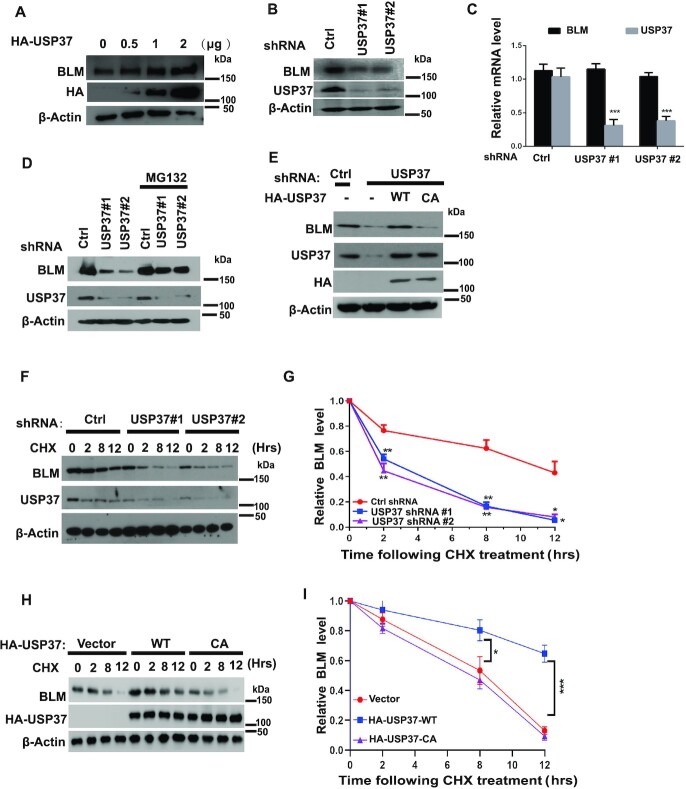
USP37 promotes BLM stabilization. (**A**) HEK293T cells were transfected with indicated dose of vector-expressing HA-USP37. After 48 h, cells were lysed, and western blot was carried out using the indicated antibodies. (**B**) USP37 shRNAs reduce BLM protein level. HEK293T cells were transduced with lentivirus carrying control (Ctrl) or USP37 shRNAs. Half of the cells were lysed and western blot was performed using the indicated antibodies. (**C**) The mRNAs were extracted from the rest of the cells from (B) and subjected to qRT-PCR. Data shown are mean ± s.d. of three independent experiments. (**D**) HEK293T cells were expressing control (Ctrl) or USP37 shRNA were treated with vehicle or MG132 for 4 h. (**E**) HEK293T cells stably expressing control (Ctrl) or USP37 shRNAs were transduced with retroviruses carrying the indicated constructs. The cells were lysed and western blot was carried out with the indicated antibodies. WT: wild type; CA: catalytically inactive mutant. (**F**, **G**) HEK293T cells stably expressing control (Ctrl) or USP37 shRNAs were treated with CHX (0.1 mg/ml) and harvested at the indicated times. Cells were lysed and cell lysates were then blotted with the indicated antibodies. (G) Quantification of the BLM protein levels relative to β-actin. Error bars represent the SEM of three independent experiments. (**H**, **I**) HEK293T cells stably expressing vector, wild type HA-USP37 or CA-HA-USP37 were treated with CHX (0.1 mg/ml) and harvested at the indicated time points. Cells were lysed and cell lysates were then blotted with the indicated antibodies. (I) Quantification of the BLM protein levels relative to β-actin. Error bars represent the SEM of three independent experiments.

We next sought to determine whether USP37 regulates BLM protein level as USP37 is a deubiquitinase. As shown in Figure 2A, overexpression of HA-tagged USP37 in HEK293T cells increased BLM protein level in a dose-dependent manner. Additionally, we depleted USP37 using two independent sets of shRNAs (short hairpin RNAs) targeting different regions of USP37 in HEK293T cells and found that USP37 knockdown markedly decreased BLM protein level but not the level of BLM mRNA (Figure 2B, C), and such effect was blocked by treatment with the proteasome inhibitor MG132 (Figure 2D). These data suggest that USP37 regulates BLM in a post-transcriptional manner and through a proteasome-mediated, protein degradation mechanism. Furthermore, reconstitution of wild-type USP37 (WT) but not the enzyme-dead mutant USP37-C350A (CA) in USP37 knockdown cells restored BLM protein levels (Figure 2E). These observations suggest that the deubiquitination activity of USP37 is essential for BLM protein level upregulation. Previous studies showed that BLM is accumulated in S phase (39). We further examined whether USP37 regulated cell cycle, which indirectly affect BLM level. However, we did not find significant changes in the cell cycle profile when we knockdown of USP37 or over-expressed of USP37 (Supplementary Figure S1A–D). In addition, overexpression of USP37 stabilized BLM in both G1 and S phase (Supplementary Figure S1E). These results suggested that the regulation of USP37 on BLM protein level was not due to an indirect effect of the change in cell cycle profile.

To further explore whether USP37 stabilizes BLM, we used cycloheximide (CHX) to inhibit protein synthesis and determined the half-life of BLM. The half-life of BLM protein dramatically decreased in USP37 knockdown cells (Figure 2F, G). On the other hand, overexpression of USP37-WT, but not the CA mutant, significantly increased BLM protein stability (Figure 2H,I). These data strongly support the notion that USP37 regulates the stability of BLM in cells.

### USP37 deubiquitinates BLM *in vivo* and *in vitro*

**Figure 3.**
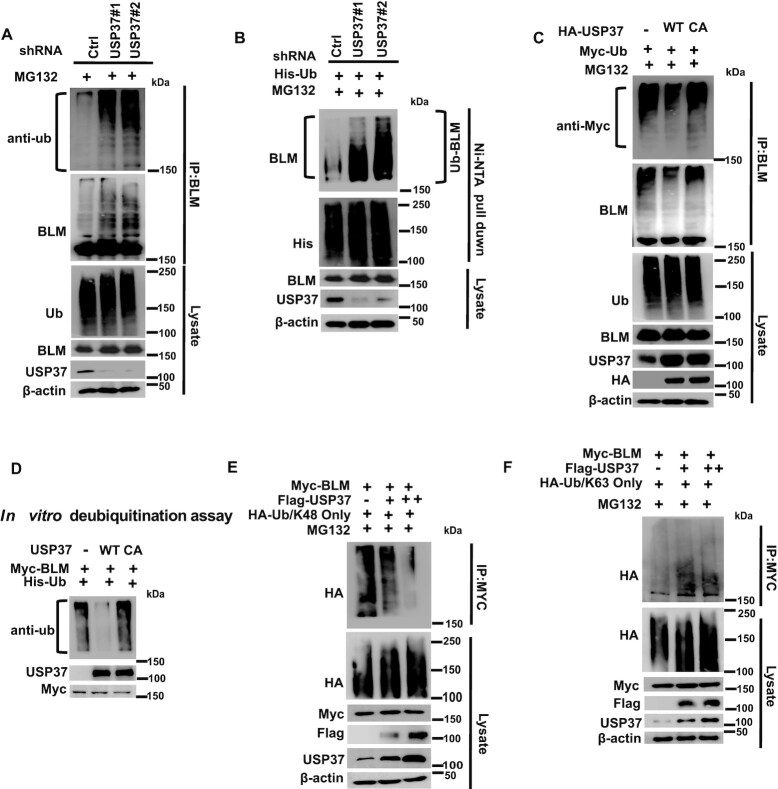
USP37 deubiquitinates BLM *in vivo* and *in vitro*. (**A**) HEK293T cells stably expressing control (Ctrl) or USP37 shRNAs were treated with MG132 for 6 h. The Ctrl cells or USP37-deficient cells were lysed under denaturing conditions, and the harvested cells were subjected to immunoprecipitation using BLM antibodies. At last the level of ubiquitin conjugates of BLM was detect by western blotting assay. (**B**) HEK293T cells stably expressing control (Ctrl) or USP37 shRNAs were treated with MG132 for 6 h before harvest and Ni-NTA beads were used to pull down His-tagged ubiquitin. Blots were probed with the indicated antibodies. (**C**) HEK293T cells stably expressing wild type USP37 (WT) or the catalytically inactive mutant (USP37C350A) were transfected with indicated plasmids. After 48 h, cells were treated with MG132 for 6h before harvest. BLM was immunoprecipitated. Blots were probed with the indicated antibodies. (**D**) Deubiquitination of BLM *in vitro* by USP37. Ubiquitinated BLM was incubated with purified USP37 WT or USP37 CA *in vitro*, and then blotted with the indicated antibodies. (**E**, **F**) HEK293T cells stably expressing Myc-BLM were cotransfected with different amounts of Flag-USP37 and HA-Ub/K48-only or HA-Ub/K63 only followed by IP analysis. Blots were probed with the indicated antibodies.

Next, we examined whether USP37 regulates the ubiquitination of BLM. As shown in Figure 3A, we found increased polyubiquitination of BLM in USP37 knockdown cells. Moreover, the polyubiquitination of BLM in USP37 knockdown cells was increased when we purified ubiquitinated proteins under denaturing conditions (Figure 3B). On the other hand, we found that USP 37-WT, but not the CA mutant, decreased BLM polyubiquitination (Figure 3C), suggesting that USP37 regulates the ubiquitination of BLM through its ubiquitin protease activity. In addition, WT USP37, but not the CA mutant, decreased BLM polyubiquitination *in vitro* (Figure 3D, and Supplementary Figure S2A, B). On the other hand, our results showed that USP37 deubiquitinated K48, but not K63 ubiquitin chains (Figure 3E, F). Overall, these data indicate that USP37 targets BLM for deubiquitination and that BLM is a bona fide substrate of USP37.

### USP37-promoted BLM stabilization is potentiated by DNA damage

**Figure 4.**
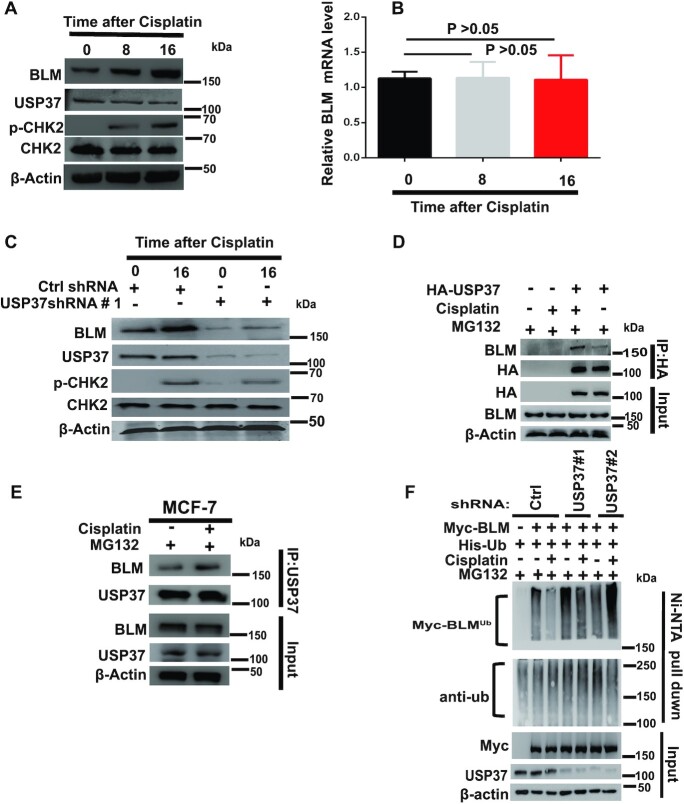
USP37-promoted BLM stabilization is potentiated by DNA damage. (**A**) HEK293T cells exposed to cisplatin (3 μmol/l) were collected at the indicated time points and analyzed by western blotting with phospho-CHK2 as a positive control. (**B**) Total RNA was collected from HEK293T cells exposed to cisplatin followed by qRT-PCR analysis of BLM expression. Data represent the mean ± SD of biological triplicate experiments. (**C**) HEK293T cells stably expressing control (Ctrl) or USP37 shRNAs were exposed to cisplatin (3 μmol/L) and collected at the indicated time points. Expression of the indicated proteins was assessed by western blotting. (**D**) HEK293T cells stably expressing vector or wild type HA-USP37 were exposed to cisplatin (3 μmol/l) for 16 h and then treated with MG132 for 4 h to normalize the BLM protein level. IP with HA-tagged beads was performed. Blots were probed with the indicated antibodies. (**E**) MCF7 cells were exposed to cisplatin (3 μmol/l) for 16 h and then treated with MG132 for 4 h to normalize the BLM protein level. Cellular extracts were immunoprecipitated and then immunoblotted with antibodies against the indicated proteins. (**F**) HEK293T cells stably expressing control (Ctrl) or USP37 shRNAs were transfected with Myc-BLM and His-Ub for 36 h, cells were then untreated or treated with cisplatin (3 μmol/l) for 16 h, and then treated with MG132 for 4 h before harvest. Cell lysates were immunoprecipitated with Ni-NTA beads. Blots were probed with the indicated antibodies.

Previous studies showed that BLM acts as an upstream sensor protein involved in the DNA damage signaling cascade (25,40). Therefore, we were interested in whether DNA-damaging agents had any influence on USP37-promoted stabilization of BLM and whether the USP37–BLM signaling pathway plays a role in the DDR. We treated HEK293T cells with cisplatin at different time points and harvested the cells. BLM protein, but not mRNA, levels increased in a time-dependent manner after cisplatin treatment (Figure 4A, B). Notably, cisplatin-induced BLM accumulation was blocked in USP37 knockdown cells (Figure 4C). These data indicate that USP37 regulates the abundance of BLM in response to DNA damage. To support this notion, we found that the interaction between USP37 and BLM was enhanced upon cisplatin treatment in cells (Figure 4D). MCF7 cells also showed similar effects (Figure 4E). Interestingly, we also found that the ubiquitination of BLM decreased following DNA damage (Figure 4F, lanes 2 and 3). In contrast, the basal ubiquitination of BLM increased in USP37 knockdown cells and did not decrease upon DNA damage (Figure 4F). Collectively, these results indicate that DNA damage can strengthen the interaction between USP37 and BLM, which in turn stabilizes BLM.

### Regulation of USP37 by DDR signaling

**Figure 5.**
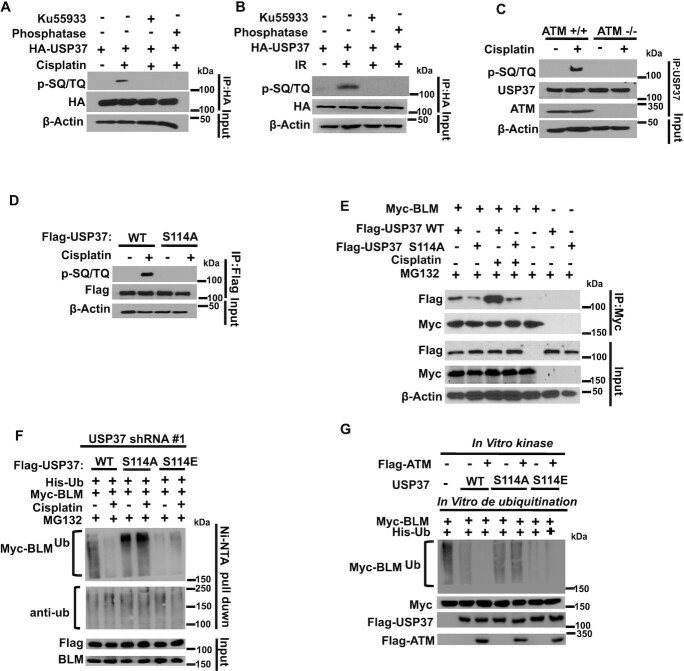
Regulation of USP37 by DDR signaling. (**A**) HEK293T cells stably expressing HA-USP37were pretreated with DMSO or 10 μM Ku55933 for 4 h followed by mock treatment or cisplatin (3 μmol/l) for 16 h. After that, HA-USP37 was immunoprecipitated, left untreated or treated with phosphatase, and immunoblotted with phospho-SQ/TQ (pSQ/TQ) antibody. (**B**) HEK293T cells stably expressing HA-USP37were pretreated with DMSO or 10 μM Ku55933 for 4 h followed by mock treatment or IR (10 Gy). After that, HA-USP37 was immunoprecipitated, left untreated or treated with phosphatase, and immunoblotted with phospho-SQ/TQ (pSQ/TQ) antibody. (**C**) ATM^+/+^ or ATM^−/−^cells were treated with cisplatin (3 μmol/l) for 16 h or left untreated. After that, USP37 was immunoprecipitated, and blots were probed with the indicated antibodies. (**D**) HEK293T cells stably expressing FLAG-USP37 (WT or S114A) were left untreated or cisplatin (3 μmol/l) for 16 h. Flag-USP37 was immunoprecipitated and blots were probed with the indicated antibodies. (**E**) HEK293T cells were transfected with the indicated plasmids and untreated or treated with cisplatin (3 μmol/l) for 16 h and MG132 for 4 h to normalize the BLM protein level and thenfollowed by co-IP and western blots. (**F**) USP37 knockdown HEK293T cells stably expressing the indicated constructs were left untreated or treated with cisplatin (3 μmol/l) for 16 h and MG132 for 4 h. Cells were lysed under denaturing conditionsand then immunoprecipitated with Ni-NTA beads and blotted with indicated antibodies. (**G**) Purified WT-USP37, the S114A or S114E mutant was incubated with ATM for *in vitro* kinase assay, and then ATM-phosphorylated USP37 constructs were used for *in vitro* deubiquitination reaction with ubiquitinated Myc-BLM. The reactions were then blotted with the indicated antibodies. WT: wild type; S114A: phosphorylation-resistant mutant; S114E: the phosphomimicking mutant.

Since USP37 inhibits the ubiquitination of BLM following DNA damage, we further explored whether and how USP37 itself is regulated following DNA damage. It is well known that the kinases ATM and ATR play important roles in regulating DNA damage signaling by phosphorylating and activating the downstream signaling networks. From analysis of ATM- and ATR-substrate proteomic data, we found that USP37 may be a potential ATM/ATR substrate (41). Our results indeed showed that following cisplatin or ionizing radiation (IR) treatment, USP37 is phosphorylated at SQ/TQ motifs (Figure 5A and B), which are consensus ATM or ATR phosphorylation sites (41). Ku55933, a specific ATM inhibitor, or lambda phosphatase could abolish this phosphorylation (Figure 5A,B). Furthermore, USP37 was phosphorylated at the SQ/TQmotif in ATM^+/+^ but not ATM^−/−^cells (Figure 5C). We also found that BLM is more stable (by CHX chase) in ATM^+/+^ cells (Supplementary Figure S3A, B). These results confirm that ATM phosphorylates USP37 following DNA damage. Through the analysis of USP37 protein sequence and public proteomic database (42), we found the only one SQ/TQ motif at S114 in USP37. We further confirmed that mutation of S114 (S114A) abrogated the phosphorylation following cisplatin treatment (Figure 5D), suggesting that USP37 is phosphorylated at S114 by ATM following DNA damage. Next, we detected whether USP37 phosphorylation has effects on its function. We found that S114A mutation attenuated the interaction between USP37 and BLM (Figure 5E). To further clarify the biological significance of USP37 phosphorylation, USP37 knockdown cells were made to stably express USP37 WT, phosphorylation-resistant S114A, or the phosphomimicking S114E mutant. As shown in Figure 5F, the ubiquitination of BLM was decreased following cisplatin treatment in knockdown cells rescued with WT USP37 but not in those rescued with the S114A mutant. Moreover, reintroduced with the phosphomimicking S114E mutant decreased the BLM polyubiquitination equally in cells with or withour cispaltin treatment (Figure 5F), suggesting that USP37 phosphorylation plays crucial roles for USP37’s deubiquitination of BLM following DNA damage. To further investigate whether USP37 phosphorylation is able to activate USP37, a two-step assay was carried out. First, we perfomed an *in vitro* kinase reaction of USP37 using ATM and we followed this with an *in vitro* deubiquitination assay. Interestingly, WT USP37 was phosphorylated by ATM, which in turn increased its deubiquitination activity towards BLM, while the S114A mutant did not have this effect. However, the phosphomimicking S114E mutant can decrease the BLM polyubiquitination equally with or without ATM phosphorylation (Figure 5G, and Supplementary Figure S3C, D). Collectively, these data suggest that S114 phosphorylation enhances the interaction between UPS37 and BLM and S114 phosphorylation of USP37 by ATM is important for USP37 deubiquitinating BLM following DNA damage.

### USP37 regulates DDR through BLM

**Figure 6.**
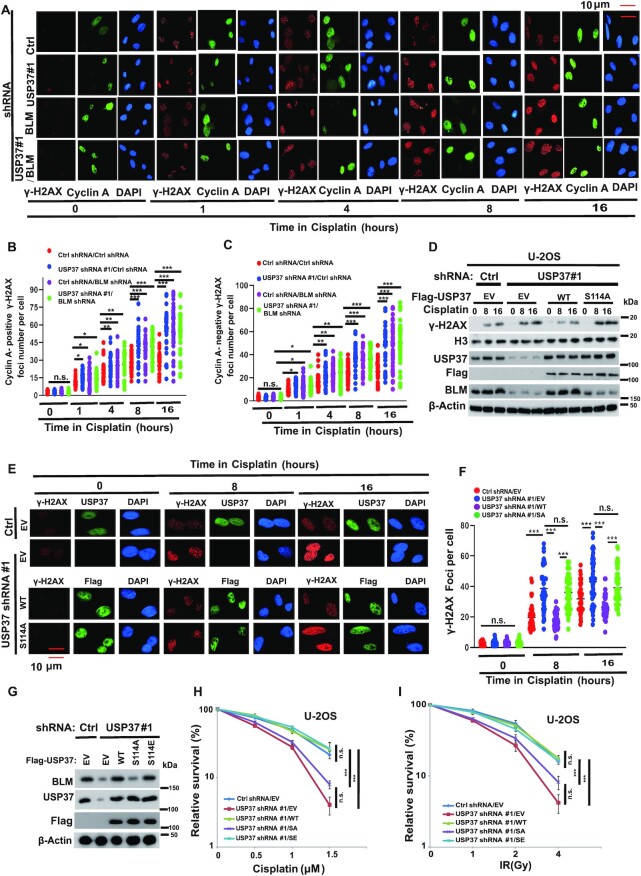
USP37 regulates DDR through BLM. (**A**) Cells from Supplementary Figure S4A were treated with cisplatin (3 μmol/l) at the indicated time points before fixing and processed for γ-H2AX immunofluorescence. Representative γ-H2AX foci (cisplatin treatment) are shown (A) (magnification: 40×). (**B**, **C**) Quantification of γ-H2AX focus formation in (A). The graph represents mean ± 95% CI, two-tailed, unpaired *t*-test was applied (*n* = 60 in each group). (**D**) USP37-WT, but not S114A mutant, rescued USP37 knockdown-mediated high levels of γ-H2AX post-cisplatin treatment at the indicated time points. IB (Immunoblot) analysis of WCLs (whole-cell lysates) derived from USP37 knockdown U-2OS cells rescued with Ctrl, USP37-WT and USP37-S114A as well as empty vector (EV), which were treated with cisplatin (3 μmol/l) at indicated time points before harvesting. (**E**) Cells from (D) treated with cisplatin (3 μmol/l) at the indicated time points before fixing and processed for γ-H2AX immunofluorescence. Representative γ-H2AX foci (cisplatin treatment) are shown (E) (magnification: 40×). (**F**) Quantification of γ-H2AX focus formation in (E). The graph represents mean ± 95% CI, two-tailed, unpaired *t*-test was applied (*n* = 60 in each group). (**G**–**I**) The response of control cells, USP37 knockdown cells and USP37 knockdown U-2OS cells stably expressing the indicated constructs to cisplatin (H) or ionizing radiation (IR) (I) was assessed using colony formation assays. USP37 expression is shown in (G). Data are the mean (±SEM) of three independent experiments. Statistical significance was assessed by two-way ANOVA with Tukey's multiple comparisons test.

Loss/mutation of BLM impaired DNA end resection and increased DNA damage that can be detected by higher levels of γ-H2AX staining (43,44). To explore the physiologic function of USP37–BLM axis in the DNA damage process, we detected γ-H2AX foci formation in USP37 or BLM knockdown cells after exposure to cisplatin (5,45). As shown in Figure 6A–C, the results revealed significantly higher γ-H2AX induction in USP37 knockdown cells after cisplatin treatment in a time dependent manner. However, depletion of USP37 did not further elevate γ-H2AX foci formation in BLM knockdown cells, suggesting USP37 regulates DDR through BLM. We stained cyclinA to represent S/G2 phase cells and we found higher γ-H2AX induction in USP37 depletion was accumulated in both cyclinA positive cells and cyclinA negative cells (Figure 6A–C). Furthermore, as shown in Supplementary Figure S4A–C, depletion of USP37 increased retention of γ-H2AX foci at at late time points in response to IR in control but not BLM knockdown cells. We next examined whether USP37–BLM axis regulates DNA end resection. As shown in Supplementary Figure S4D-L, we performed the DSB end resection assays in ER-AsiSI U2OS cells. We found that depleted of USP37 decreased the DNA end resection. However, reconstituting USP37 knockdown cells with USP37-WT, but not the USP37-CA mutant, rescued these phenotypes (Supplementary Figure S4E,F). Furthermore, we found that depleted of USP37 decreased DSB end resection in control cells but not BLM depletion cells (Supplementary Figure S4G, H). In addition, over-expression of BLM rescued DSB end resection in USP37 depletion cells (Supplementary Figure S4I, J). Moreover, over-expression of USP37 increased DSB end resection in control cells but not BLM depletion cells (Supplementary Figure S4K, L). These results suggested that USP37 regulates DNA end resection in a BLM dependent manner. The hallmark features of BLM-deficient cells also include elevated frequency of ultra-fine anaphase bridges (UFBs) (46) and sister chromatid exchanges (SCEs) (39). As shown in Supplementary Figure S4M-R, depleted of USP37 increased the frequency of UFBs (Supplementary Figure S4M-O) and SCEs (Supplementary Figure S4P-R) in control cells but not BLM depletion cells. Taken all together, these data suggested that USP37 regulates DDR in a BLM dependent manner.

To further demonstrate a potential function of USP37 phosphorylation, we reintroduced USP37 knockdown cells with WT USP37 or the USP37 S114A mutant. Notably, we found that reconstituted USP37 WT, but not S114A, could rescue the elevated γ-H2AX status (DNA damage) upon cisplatin treatment (Figure 6D–F). Moreover, as shown in (Figure 6G–I and Supplementary Figure S5A–H), depleted of USP37 led cells sensitize to cisplatin or IR and decreased DNA end resction. However, reconstituted of USP37 knockdown cells with USP37WT or S114E but not the USP37-S114A mutant rescued this phenotype. Together, these data suggest that USP37 phosphorylation by ATM is important for its regulation of DDR.

### USP37 regulates chemotherapy and radiotherapy resistance via BLM

**Figure 7.**
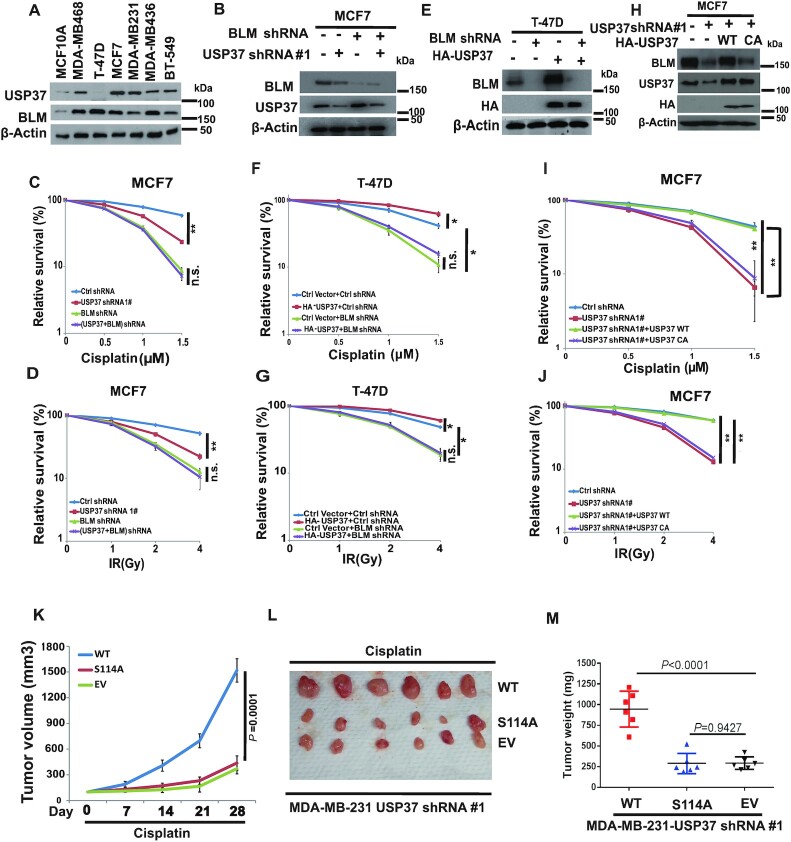
USP37 regulates chemotherapy and radiotherapy resistance via BLM. (**A**) Immunoblot of indicated proteins in human breast epithelial cell line and breast carcinoma cell lines. (**B**) MCF7 cells stably expressing indicated constructs were lysed. Cells were lysed and cell lysates were blotted with the indicated antibodies. (C, D) Cells from (B) were treated with (**C**) cisplatin or (**D**) IR. Cell survival was performed by CCK8 assays. (**E**) T-47D cells stably expressing indicated constructs were lysed. Cells were lysed and cell lysates were blotted with the indicated antibodies. (F–G) Cells from (**E**) were treated with (**F**) cisplatin or (**G**) IR. Cell survival was performed by CCK8 assays. (**H**) MCF7 cells stably expressing indicated constructs were lysed. Cells were lysed and cell lysates were blotted with the indicated antibodies. (I, J) Cells from (H) were treated with (**I**) cisplatin or (**J**) IR. Cell survival was performed by CCK8 assays. The data presented are mean ± SD (*n* = 6). (K–M) Tumor xenograft assays were performed by subcutaneous injection of USP37 knockdown MDA-MB-231 cells stably expressing USP37 WT, S114A, and EV. Tumor growth rate in nude mice treated every other day with cisplatin (20 mg/kg) is shown (**K**). Tumors were dissected and recorded after euthanizing the mice (**L**). Mice were sacrificed after 28 days. Tumor weights were measured as shown in (**M**). Representative data (mean ± SEM) are shown from six mice each group by two-sided unpaired *t*-test.

It is well known that the status of the DDR pathway has an effect on cancer cell response to chemotherapy or radiotherapy, and loss of DDR elements induces sensitivity to DNA-damaging agents (47). We next examined the role of the USP37–BLM axis in the response to chemo or radiotherapy in human cancer cells. Previous publication showed that BLM is widely expressed in tumour tissue of both lymphoid and epithelial origin (48). We tested the expression profile of USP37 and BLM in normal breast epithelium and a number of breast cancer cell lines, and found that both of them were over-expressed in the breast cancer cell lines (Figure 7A). However, the S-phase fractions of these cancer cells are similar to the normal cell line MCF10A (Supplementary Figure S6A). To further investigate the potential role of the USP37–BLM axis as a target for cancer therapy, we knocked down USP37 or BLM individually or together in MCF7 cells or MDA-MB-231 cells with high USP37 level (Figure 7B), and examined the cell survival following cisplatin or IR treatment. We found that knockdown of USP37 rendered MCF7 and MDA-MB-231 cells sensitive to cisplatin or IR (Figure 7C, D and Supplementary Figure S6B–D). However, knocking down USP37 in BLM depleted cells did not further sensitize cells to these treatments. In addition, overexpression of USP37 in T-47D cells, with low USP37 expression, rendered cells resistant to cisplatin or IR treatment (Figure 7E–G). Conversely, overexpression of USP37 does not confer resistance to cisplatin or IR in BLM depleted cells (Figure 7F, G). To further confirm that the regulation of resistance to chemotherapy or radiotherapy by USP37 is dependent on its catalytic activity, we reconstituted USP37 knockdown cells with USP37-WT or USP37-C350A mutant (Figure 7H and Supplementary Figure S6E) and examined the cell survival upon cisplatin or IR treatment. As shown in Figure 7I,J and Supplementary Figure S6F,G, downregulation of USP37 sensitized cells to cisplatin or IR. However, reconstitution of WT USP37 but not the C350A mutant restored these phenotypes, suggesting that the catalytic activity of USP37 is important for its regulation of the cellular response to cisplatin or IR treatment. Moreover, we found that overexpressed BLM restored the cells resistance to cisplatin or IR in the USP37 knockdown cells reconstituted with USP37-C350A mutant (Supplementary Figure S6H–J). Taken together, these results suggest that USP37 regulates cellular response to cisplatin or IR in breast cancer cells in a BLM-dependent manner.

Next, we further investigated USP37 as a potential target for breast cancer therapy *in vivo*. As shown in Supplementary Figure S6K, the reconstituted USP37 expression levels are almost equal to endogenous USP37. Next, we analyzed the cell proliferation in these cells with or without cisplatin treatment and found reconstituted with USP37 WT but not S114A (phosphorylation-deficient) mutant led cancer cell resistant to cisplatin treatment *in vitro* (Supplementary Figure S6L). In addition,as shown in Figure 7K–M, reconstitution of USP37 WT but not the S114A mutant, cause cellular resistance to cisplatin treatment in the xenograft model. We also analyzed the xenografts tumour tissues and found that the BLM level is higher in the USP37 WT tumors compare with USP37 S114A tumors (Supplementary Figure S6M). Taken together, these data suggests that USP37 plays a critical role in cellular response to DSB-inducing agents.

### USP37 expression positively correlates with BLM levels in breast cancer tissues

**Figure 8.**
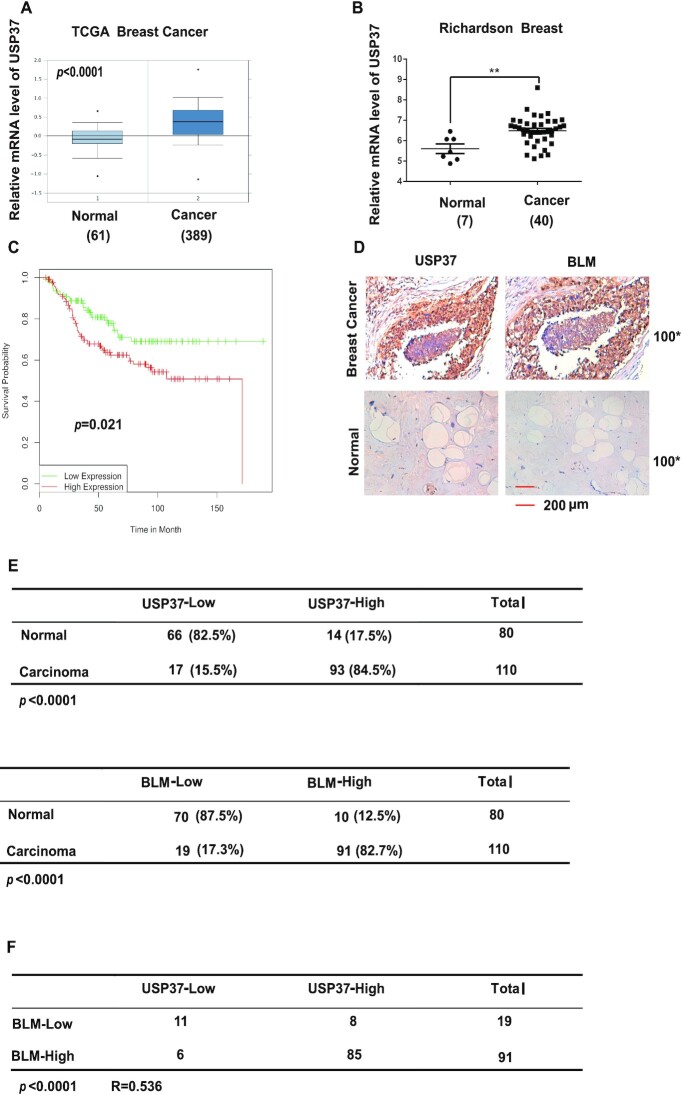
USP37 expression positively correlates with BLM levels in breast cancer tissues. (**A**, **B**) USP37 expression in normal tissue and breast carcinoma (Oncomine data). (**C**) GEO database showing the survival of breast cancer patients with low (*n* = 112) and high (*n* = 127) expression of USP37. Statistical analysis with the two-sided log-rank (Mantel-Cox) test revealed statistically significant differences as shown on the graph. (**D**) Representative images of immunohistochemical staining of USP37 and BLM in normal and breast carcinoma. Scale bars, 200 μm. (**E**) Quantification of USP37 and BLM protein levels in normal and breast carcinoma. (**F**) Correlation study of USP37 and BLM in breast carcinoma. Statistical analyses were performed with the *χ*^2^ test. *R*: the Pearson correlation coefficient.

Previous studies showed that higher DDR mediated resistance to chemo-or radiotherapy in cancer (49). Up to this point, we determined that USP37 overexpressed in breast cancer cell lines and overexpression of USP37 lead breast cancer cells to be more resistant to chemo or radiotherapy (Figure 7 and Supplementary Figure S6). To further determine the clinical relevance between USP37–BLM axis and breast cancer, we examined the expression of USP37 and BLM in breast cancer samples. As shown in Figure 8A, B, we found that USP37 is over-expressed in human breast carcinoma by surveying a public gene expression database (http://www.oncomine.org). Moreover, clinical data from the GEO database indicated that increased expression of USP37 was markedly correlated with poor survival of breast cancer patients (Figure 8C). Next, we detected the expression of USP37 and BLM in human breast cancer samples by immunohistochemical (IHC) analyses. The specificity of anti-USP37 antibody was shown in Supplementary Figure S7A-C. As shown in Figure 8D, E, up-regulation of USP37 was detected in 84.5% (93 of 110) of breast cancer tissues, while 82.5% (66 of 80) of adjusted normal breast tissues showed low expression of USP37, confirming that USP37 is overexpressed in human breast cancer samples. In addition, BLM expression is also up-regulated in human breast cancer tissues (Figure 8D, E). Moreover, a positive correlation between BLM and USP37 protein levels (*P* < 0.0001, *R* = 0.536) was observed in these breast carcinomas (Figure 8F). Collectively, these data suggest that overexpression of USP37 correlates with up-regulation of BLM in human breast cancer tissues. In addition, GEO database showed that high expression of USP37 is related to poor survival in breast cancers.

In summary, BLM, a DNA helicase, is important for the DNA damage response. BLM is overexpressed in multiple cancers, including breast cancer, however, the mechanism is not clear. In this study, we demonstrate that USP37 deubiquitinates and stabilizes BLM. Mechanistically, DNA damage activates ATM which phosphorylates USP37 and activates its deubiquitinase activity to stabilize BLM. In addition, USP37 is overexpressed in breast cancer samples, and is correlated with poor survival. Moreover, a positive correlation between BLM and USP37 protein levels was observed in the breast carcinomas. Finally, we demonstrated that high level of USP37 mediated cisplatin or IR resistance in breast cancer in a BLM-dependent manner and clarified the role of USP37 as a potential therapeutic target in breast cancer.

## DISCUSSION

Endogenous and exogenous agents induce DNA damage and impair genome integrity. Therefore, timely and accurate DNA repair plays an important role in maintaining genome integrity and human health, as genome instability is closely linked to cancer predisposition and aging. The RecQ family of DNA helicases are involved in error-free DNA damage repair to preserve genome integrity. There are five family members including RECQ1, BLM, WRN, RECQ4 and RECQ5 in humans. Mutations of BLM in humans lead to Bloom syndrome (BS), which can result in immunodeficiency, dwarfism, sterility, premature aging, and multiple cancers, such as breast cancer (50). BLM unwinds specific DNA structures including D-loops, forked duplexes, G-quadruplex (G4) DNA and Holliday junctions through its 3′-to-5′ DNA helicase activity (51–54) and plays pivotal roles in multiple repair pathways that maintain genome stability and prevent cancer. BLM binds with RMI1, RMI2 and topoisomerase IIIα to form a BTR complex, dissolving the Holliday junction, thereby inhibiting crossover and sister chromatin exchange (55,56). BLM is responsible for ensuring complete sister chromatid decatenation and plays key roles in the inhibition of anaphase bridges and faithful chromosome segregation (46). BLM is also an upstream sensor protein, which plays key roles in DNA damage signaling cascade (25,40). Previous publications also showed that BLM plays important roles at stalled replication forks (57,58). BLM was reported to promote DNA repair synthesis in D-loops and supports Homologous recombination (HR) (43,59). On the other hand, BLM plays a vital role in disruption of RAD51filament formation, the disruption of D-loops and the dissolution of double Holliday junctions to oppose homologous recombination (60,61). Therapeutic targeting of DNA damage repair enzymes have recently shown promise as a chemotherapeutic strategy due to increased genomic instability in cancer (62). The BLM helicase inhibitor ML216 has been applied to modulate BLM helicase chromosome stability in human cells (63). However, this compound lacked specificity and also targeted or suppressed the ability of closely associated helicases to unwind DNA, resulting in off-target effects (63,64). Therefore, new biomarkers in cancers associated with BLM need to be discovered. Whether and how the BLM is regulated upon DDR is still unclear.

Post-translational modification (PTM) of BLM is important for its function in DNA repair and tumor radiosensitivity. Phosphorylation, SUMOylation, and ubiquitination of BLM have been reported, in turn regulating its subcellular localization, protein/protein interactions, and protein stability (65). For example, ATM and ATR are known to phosphorylate BLM on Thr99 and Thr122 following irradiation and during replication stress, respectively (66,67). Chk1 and Chk2 are reported to phosphorylate BLM at serine 646 in response to DNA damage, which contributes to BLM’s relocalization to damage sites (40,68). Additionally, two studies show that BLM modification by SUMO plays vital roles on the induction of repair foci as well as altering protein subcellular localization to promyelocytic leukemia nuclear bodies (PML-NBs) (69,70). The Mindbomb 1 (MIB1) E3 ligase is reported to degrade BLM protein levels by the 26S proteasome upon ubiquitination in G1-phase (39). However, the role of deubiquitination of BLM in DDR remains unknown. Here, we identified the deubiquitinase, USP37, that directly deubiquitinates and stabilizes BLM in response to DNA damage.

Previous studies showed that USP37 regulates diverse cellular events by mediating the deubiquitination of various target proteins. For example, USP37-mediated deubiquitinaition of Wings Apart-Like (WAPL) affected chromosomal cohesion and mitotic progression (71). Other work shows that USP37 stabilizes c-Myc in lung cancer, promoting lung cancer cell proliferation (72). USP37 was also reported to remove ubiquitin chains from Cyclin A and overexpression of USP37 promotes Cyclin A accumulation and accelerated S phase entry, suggesting a potentially key role for USP37 in regulating the cell cycle in a cancer setting (73).

In this study, we carried out a screen for potential BLM DUBs and found that USP37 interacts with BLM, leading to deubiquitination and stabilization of BLM, which in turn regulates the DNA Damage Response (DDR) in the BLM-dependent manner. Future studies will be needed to investigate the physiological significance of USP37 stabilization of BLM *in vivo*.

Previous study showed that depletion of USP37 reduces homologous recombination (HR) efficiency by promoting excessive spreading of Ub-K63 signal that promotes sequestration of BRCA1 by RAP80 (74). As shown in Supplementary Figure S8A-B, depleted of USP37 decreased DSB end resection in both control and RAP80 knockdown cells. These results suggested that besides Ub-H2A/RAP80 axis, USP37 may also regulate DDR through other substrate. Furthermore, we found that overexpression of BLM rescued DSB end resection in USP37 depletion cells (Supplementary Figure S4I, J). In addition, overexpression of BLM restores the cells resistance to cisplatin or IR treatment in USP37CA reconstitution cells (Supplementary Figure S6H–J). These results suggested that USP37 may regulates DSB end resection and cellular response to DNA damage in a BLM dependent manner. The work by Typas *et al.* (74) suggested that two DUBs, USP26 and USP37 deubiquitinate H2A and subsequent impact on DSB repair through RAP80. Some reports showed that several other DUBs such as USP51 (75) and USP16 (76) also deubiquitinate H2A and regulate DDR. Since USP37 is one of the H2A DUBs, compared to USP37 mediated H2A deubiquitination, USP37 mediated BLM deubiquitination and stabilization may also have key function in DNA damage response. Therefore, in our study, we added additional layer of DDR regulation through USP37–BLM axis besides the USP37 deubiquitinating H2A.

In addition, USP37 is overexpressed in breast cancer samples and GEO database showed that high expression of USP37 is related to poor survival in breast cancers. Moreover, a positive correlation between BLM and USP37 protein levels was observed in the breast carcinomas supporting a role for the activity of the USP37–BLM axis in human breast cancer cells. It will be interesting to further investigate the molecular mechanisms underlying the overexpression of the USP37–BLM axis in breast cancer. Nevertheless, our results reveal that USP37 regulates the stability, ubiquitination and thus the function of BLM, providing a mechanistic link between the deubiquitinase USP37 and the BLM-mediated DDR. Our findings indicate that the USP37–BLM axis plays a critical role in the cellular response to cisplatin or IR treatment in breast cancer, supporting the pursuit of molecules targeting this pathway in breast cancer intervention. Therefore, targeting USP37 in the breast cancer may sensitize cancer to cisplatin or IR treatment. Finally, considering that USP37 is overexpressed in breast cancer, small molecules that inhibit USP37 have broad development prospects and a combination of USP37 inhibitor with platinum-based therapy or IR may provide a novel approach for breast cancer therapy.

## Supplementary Material

gkab842_Supplemental_Files
