# Correction to ‘USP37 regulates DNA damage response through stabilizing and deubiquitinating BLM’

**DOI:** 10.1093/nar/gkad070

**Published:** 2023-02-06

*Nucleic Acids Research*, Volume 49, Issue 19, 8 November 2021, Pages 11224–11240, https://doi.org/10.1093/nar/gkab842

The authors wish to correct several errors in Figures 6A, 7L and Supplementary Figure 4B. The errors were introduced during image assembly.

Figure 6A: Panel in the BLM knockdown condition treated with Cisplatin for 1h was accidently duplicated from panel Ctrl condition treated with Cisplatin for 4h. The image has been substituted for the corrected one in the new Figure 6A provided below. A revised Figure 6 is provided in the Supplementary data.

Figure 7L: Multiple different xenograft experiments were performed at the same time. The image of a different xenograft model was inadvertently used instead of WT, S114A and EV. The image has been substituted for the corrected one in the new Figure 7L provided below. A revised Figure 7 is provided in the Supplementary data.

Supplementary Figure 4B: Several images were accidently duplicated. The images have been substituted for the corrected ones in the new Figure S4B provided below. A revised Supplementary Figure 4B is provided in the Supplementary data.

These corrections do not affect the interpretation of the results and conclusions of the article.

## Supplementary Material

gkad070_Supplemental_File

