# Supplementary material for: USP37 regulates DNA damage response through stabilizing and deubiquitinating BLM
Source: Nucleic Acids Res. 2021 Oct 4;49(19):11224–40. doi: 10.1093/nar/gkab842 (PMC8565321; doi:10.1093/nar/gkab842)

Supplementary Figure Legends

Supplementary Figure S1. USP37 does not affect the the cell cycle distribution of the cell population.

(A) U-2OS cells were transduced with lentivirus carrying control (Ctrl), USP37 shRNAs. Cells were lysed, and western blot was carried out using the indicated antibodies.

(B) The cell cycle profiles of the control, USP37 knockdown cells from (A) were analyzed by flow cytometry.

(C) U-2OS cells were transduced with lentivirus carrying Empty Vector (EV) or Flag-USP37. Cells were lysed, and western blot was carried out using the indicated antibodies.

(D) The cell cycle profiles of t EV or Flag-USP37 cells from (C) were analyzed by flow cytometry.

(E) HEK293T cells transfected with HA-USP37 were synchronized in G1 phase by double thymidine block or released into S phase. Then the cells were treated with or without cisplatin (3μmol/L) for 1h and cell lysates were then blotted with the indicated antibodies.

Supplementary Figure S2. USP37 deubiquitinates BLM *in vitro*.

(A-B) Deubiquitination of BLM *in vitro* by USP37. Ubiquitinated BLM was incubated with purified USP37 WT or USP37 CA *in vitro,* and then blotted with the indicated antibodies. WT: wild type; CA: catalytically inactive mutant.

Supplementary Figure S3. S114 phosphorylation of USP37 by ATM is important for USP37 activation.

(A) $ATM^{+/+}$ or $ATM^{-/-}$cells were treated with CHX (0.1 mg/ml) and harvested at the indicated times. Cells were lysed and cell lysates were then blotted with the indicated antibodies.

(B) Quantification of the BLM protein levels relative to β-actin. The graph represents

mean ± S.D., two-tailed, paired t-test; n = 3 independent experiments.

(C-D) Purified WT-USP37, the S114A or S114E mutant was incubated with ATM at 30 ℃ in buffers containing [25 mM tris HCl (pH 7.4), 2 mM adenosine 5′-triphosphate (ATP), 5 mM MgCl2, 5 mM MnCl2, and 0.1 mM dithiothreitol (DTT)] for *in vitro* kinase assay, and then ATM-phosphorylated USP37 constructs were used for *in vitro* deubiquitination reaction with ubiquitinated Myc-BLM. The reactions were then blotted with the indicated antibodies. WT: wild type; S114A: phosphorylation-resistant mutant; S114E: the phosphomimicking mutant.

Supplementary Figure S4. USP37 promotes end resection, inhibits ultra-fine anaphase bridges (UFBs) and sister chromatid exchanges (SCEs) in a BLM-dependent manner.

(A) U2OS cells stably expressing the indicated shRNAs were lysed and cell lysates were then blotted with the indicated antibodies.

(B) Cells from (A) were treated with IR followed by immunofluorescent cytochemistry (IFC) of γ-H2AX foci at the indicated time points after IR (B). (Magnification: 40 ×). Scale bars in images: 10μm.

(C) Quantification of γH2AX foci in (B) cells. The graph represents mean ± 95% CI, two-tailed, unpaired t-test was applied (n=60 in each group). n.s.: not significant, **, $p<0.01$, ***, $p<0.001$.

(D) Illustration of the designation of Taqman qPCR primers and probes (black arrows) for detecting resection at sites adjacent to the AsiSI sites (red arrows). The primer pairs are across BsrGI restriction sites. All Taqman probes are designed at either side of the restriction site.

(E) ER-*AsiS*I U-2OS cells stably expressing control (Ctrl) or USP37 shRNAs were transduced with retroviruses carrying the indicated constructs. The cells were lysed and western blot was carried out with the indicated antibodies. WT: wild type; CA: catalytically inactive mutant. Blots were quantiied using Image J.

(F) The genomic DNA from (E) cells, treated with 2 μM 4-OHT for 4 h or mock treated, was then extracted and mock digested or digested with BsrGI overnight. DNA end resection adjacent to DNA double-strand break sites was measured by qPCR assay. Data are presented as mean values ± SEM from three independent experiments.

(G) ER-*AsiS*I U-2OS cells stably expressing the indicated shRNAs were lysed and cell lysates were then blotted with the indicated antibodies.

(H) The genomic DNA from (G) cells, treated with 2 μM 4-OHT for 4 h or mock treated, was then extracted and mock digested or digested with BsrGI overnight. DNA end resection adjacent to DNA double-strand break sites was measured by qPCR assay.

(I) ER-*AsiS*I U2OS cells stably expressing USP37 shRNAs and Flag-BLM were lysed and cell lysates were blotted with the indicated antibodies.

(J) The genomic DNA from (I) cells, treated with 2 μM 4-OHT for 4 h or mock treated, was then extracted and mock digested or digested with BsrGI overnight. DNA end resection adjacent to DNA double-strand break sites was measured by qPCR assay.

(K) ER-*AsiS*I U2OS cells stably expressing indicated constructs were lysed. Cells were lysed and cell lysates were blotted with the indicated antibodies.

(L) The genomic DNA from (K) cells, treated with 2 μM 4-OHT for 4 h or mock treated, was then extracted and mock digested or digested with BsrGI overnight. DNA end resection adjacent to DNA double-strand break sites was measured by qPCR assay. Quantified data In (F), (H), (J) and (L) represent the mean ± SD of n = 3 independent experiments, two-tailed, unpaired t-test.

(M) U-2OS cells stably expressing control (Ctrl) or USP37 shRNA were transduced with lentiviruses encoding control or BLM shRNA to generate stable cell lines. Cells were lysed and cell lysates were blotted with the indicated antibodies.

(N) The cells from (M) were treated with cisplatin ((3μmol/L) for 1 h and released into fresh media for 24 h. BLM was visualized using anti-BLM antibody. Deconvoluted images are shown (N). (Magnification: 40 ×). Scale bars, 10μm.

(O) Quantification of cells (60 cells per condition) with BLM-positive UFBs (ultra-fine anaphase bridges) as visualized in (N). In (N) representative images of three independent experiments are shown. P values were determined using a two-tailed-unpaired t-test. Quantified data In (O) represents the mean ± SD of n = 3 independent experiments.

(P) Representative immunoblotting of Hela cells transfected with indicated siRNAs.

(Q) USP37 suppresses SCE via BLM. Representative metaphase spreads showing SCEs from HeLa cells transfected with indicated siRNAs (red arrows). (Magnification: 60 ×.) Ctrl:Control, UT: untreated, Scale bars, 5μm.

(R) A histogram summarizing sister chromatid exchanges (SCEs) frequency of each metaphase spreads. SCEs were scored for 30 metaphase spreads for each group.

Student's *t-test* (two-tailed unpaired). n.s.: not significant, ***, $p<0.0001$

Supplementary Figure S5. USP37 phosphorylation by ATM is important for its regulation of DDR

(A) MCF7 cells stably expressing control (Ctrl) or USP37 shRNAs were transduced with lentivirus carrying the indicated constructs. The cells were lysed and western blot was carried out with the indicated antibodies.

(B-C) Cells from (A) were treated with (B) cisplatin or (C) IR. Cell survival was performed by clonogenic survival assays.

(D) U-2OS cells stably expressing control (Ctrl) or USP37 shRNAs were transduced with lentivirus carrying the indicated constructs. The cells were lysed and western blot was carried out with the indicated antibodies.

(E-F) Cells from (D) were treated with (E) cisplatin or (H) IR. Cell survival was performed by using CCK8 assay. The data presented are mean ± SD (n= 6).

(G) ER-*AsiS*I U2OS cells stably expressing control (Ctrl) or USP37 shRNAs were transduced with lentivirus carrying the indicated constructs. The cells were lysed and western blot was carried out with the indicated antibodies. WT: wild type; SA: phosphorylation-resistant S114A mutant. SE: phosphomimicking S114E mutant.

(H) The genomic DNA from (G) cells, treated with 2 μM 4-OHT for 4 h or mock treated, was then extracted and mock digested or digested with BsrGI overnight. DNA end resection adjacent to DNA double-strand break sites was measured by qPCR assay.

Supplementary Figure S6. USP37 regulates chemotherapy or radiotherapy resistance via BLM and USP37 phosphorylation by ATM is important for its regulation of chemotherapy or radiotherapy.

(A) The cell cycle profiles of the human breast epithelial cell line and breast carcinoma cell lines were analyzed by flow cytometry.

(B) MDA-MB-231 cells stably expressing control (Ctrl) or USP37 shRNA were transduced with lentiviruses encoding control or BLM shRNA to generate stable cell lines. Cells were lysed and cell lysates were blotted with the indicated antibodies.

(C-D) Cells from (B) were treated with (C) cisplatin or (D) IR. Cell survival was performed by clonogenic survival assays. Experiments were done in triplicates. Results shown are averages of three independent experiments.

(E) U-2OS cells stably expressing control (Ctrl) or USP37 shRNAs were transduced with retroviruses carrying the indicated constructs. The cells were lysed and western blot was carried out with the indicated antibodies. WT: wild type; CA: catalytically inactive mutant.

(F-G) Cells from (E) were treated with (F) cisplatin or (G) IR. Cell survival was performed by clonogenic survival assays. Experiments were done in triplicates. Results shown are averages of three independent experiments.

(H) MCF7 cells stably expressing control (Ctrl) or USP37 shRNAs were transduced with lentivirus / retroviruses carrying the indicated constructs. The cells were lysed and western blot was carried out with the indicated antibodies. Blots were quantified using Image J.

(I-J) Cells from (H) were treated with (I) cisplatin or (J) IR. Cell survival was performed by clonogenic survival assays. Experiments were done in triplicates. Results shown are averages of three independent experiments.

(K) MDA-MB-231 cells stably expressing control (Ctrl) or USP37 shRNAs were transduced with lentivirus carrying the indicated constructs. The cells were lysed and western blot was carried out with the indicated antibodies.

(L) Cells from (K) was measured for cell proliferation. Data are presented as the mean ± SD (n=6) for cell viability assays.

(M) Immunoblot of tissue lysates from the first four mice in Figure 7L. EV: empty vector.

Supplementary Figure S7. The specificity of anti-USP37 antibody was detected in MCF7 cells.

(A) MCF7 cells were transduced with lentivirus carrying control (Ctrl), USP37 shRNAs or Flag-USP37. Half of the cells were lysed and western blot was performed using the indicated antibodies.

(B) Control, USP37 knockdown or USP37-overexpressed cells from the rest of (A) were embedded and subjected to identical immunohistochemistry (IHC) staining conditions. Scale bars in images: 10μm.

(C) Quantification of (B) and the results are presented as the percentage of the control (Ctrl) (n = 3). Data are presented as the mean ± standard errors of the mean (SEM). n.s.: not significant, ***p* < 0.01; Student's *t-tes*t (two-tailed unpaired).

Supplementary Figure S8. USP37 and RAP80 act in different pathways in regulating DNA end resection.

(A) ER-*AsiS*I U2OS cells stably expressing the indicated shRNAs were lysed and cell lysates were then blotted with the indicated antibodies.

(B) The genomic DNA from (A) cells, treated with 2 μM 4-OHT for 4 h or mock treated, was then extracted and mock digested or digested with BsrGI overnight. DNA end resection adjacent to DNA double-strand break sites was measured by qPCR assay.

Supplementary Figure S1

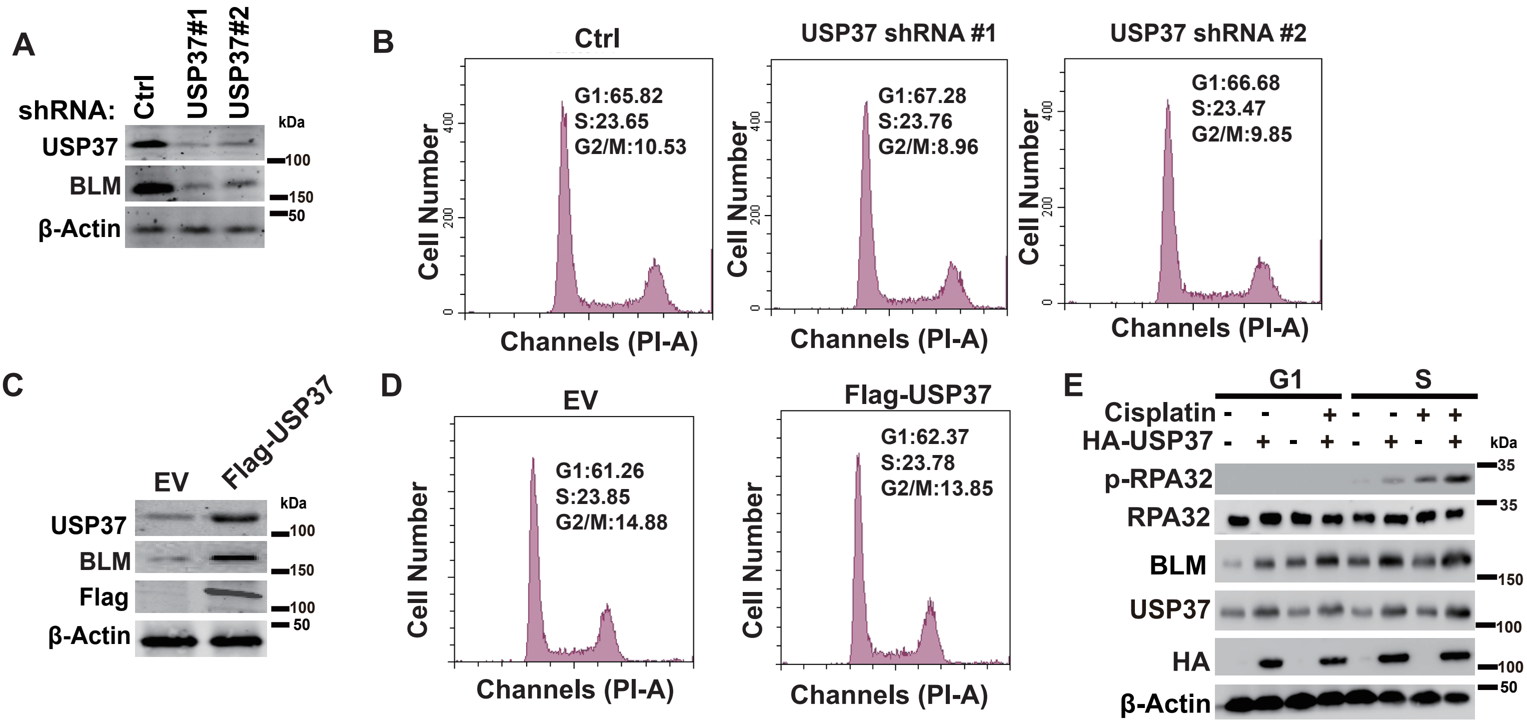

Supplementary Figure S2

A

***In vitro* deubiquitination assay**

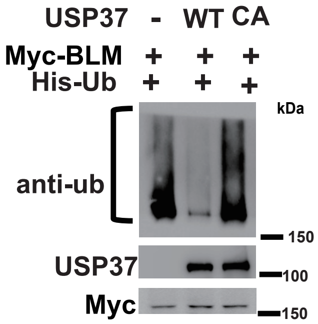

B

***In vitro* deubiquitination assay**

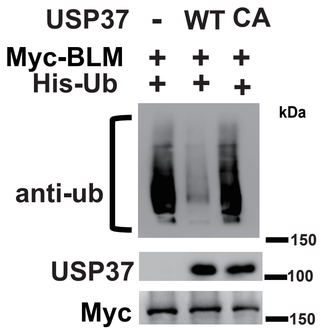

Supplementary Figure S3

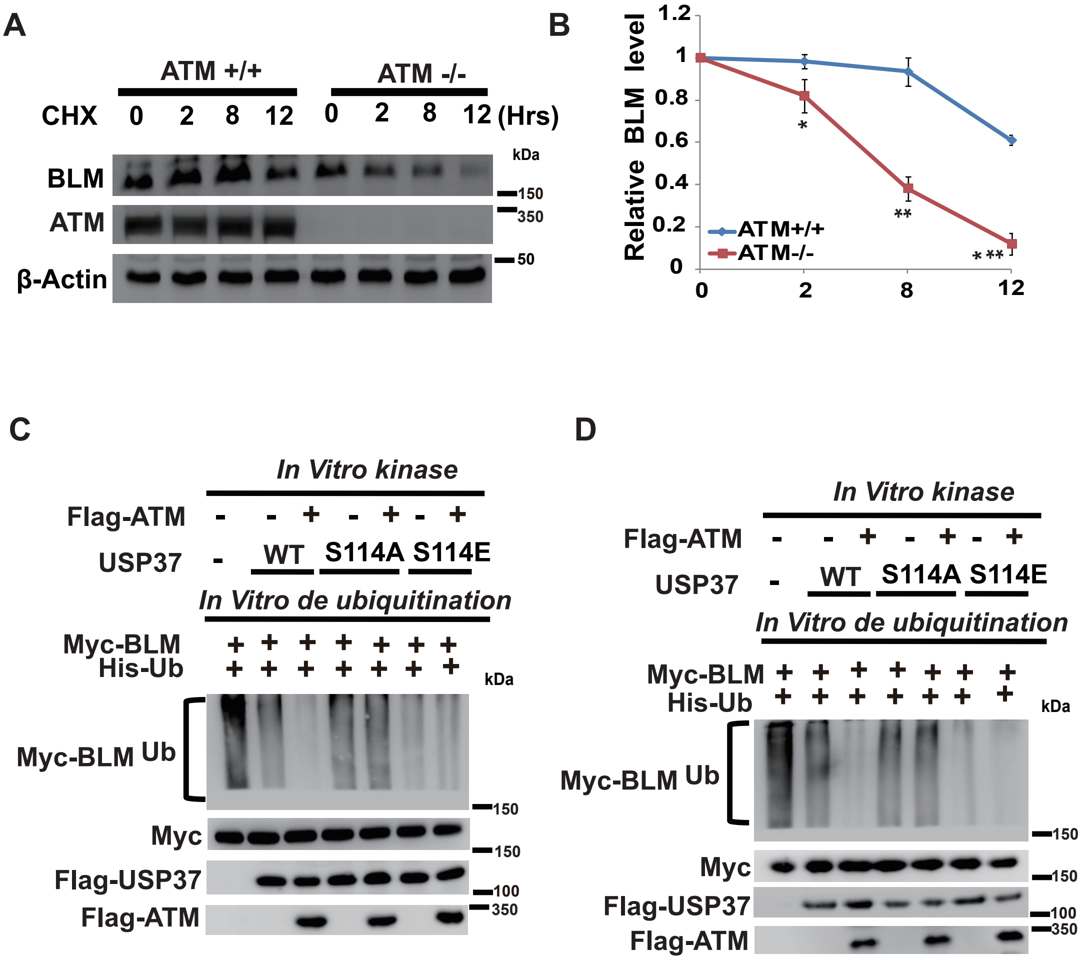

Supplementary Figure S4

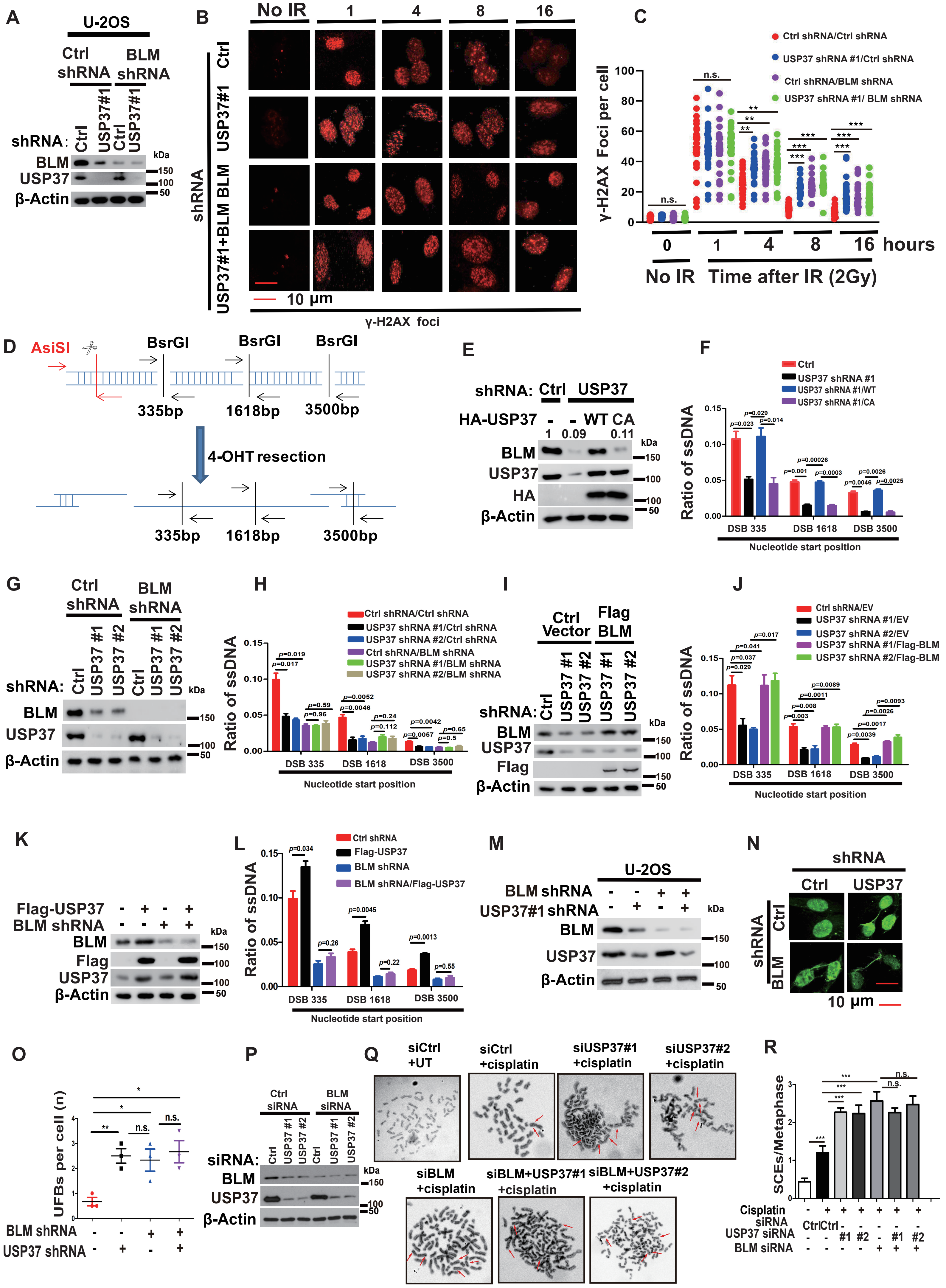

Supplementary Figure S5

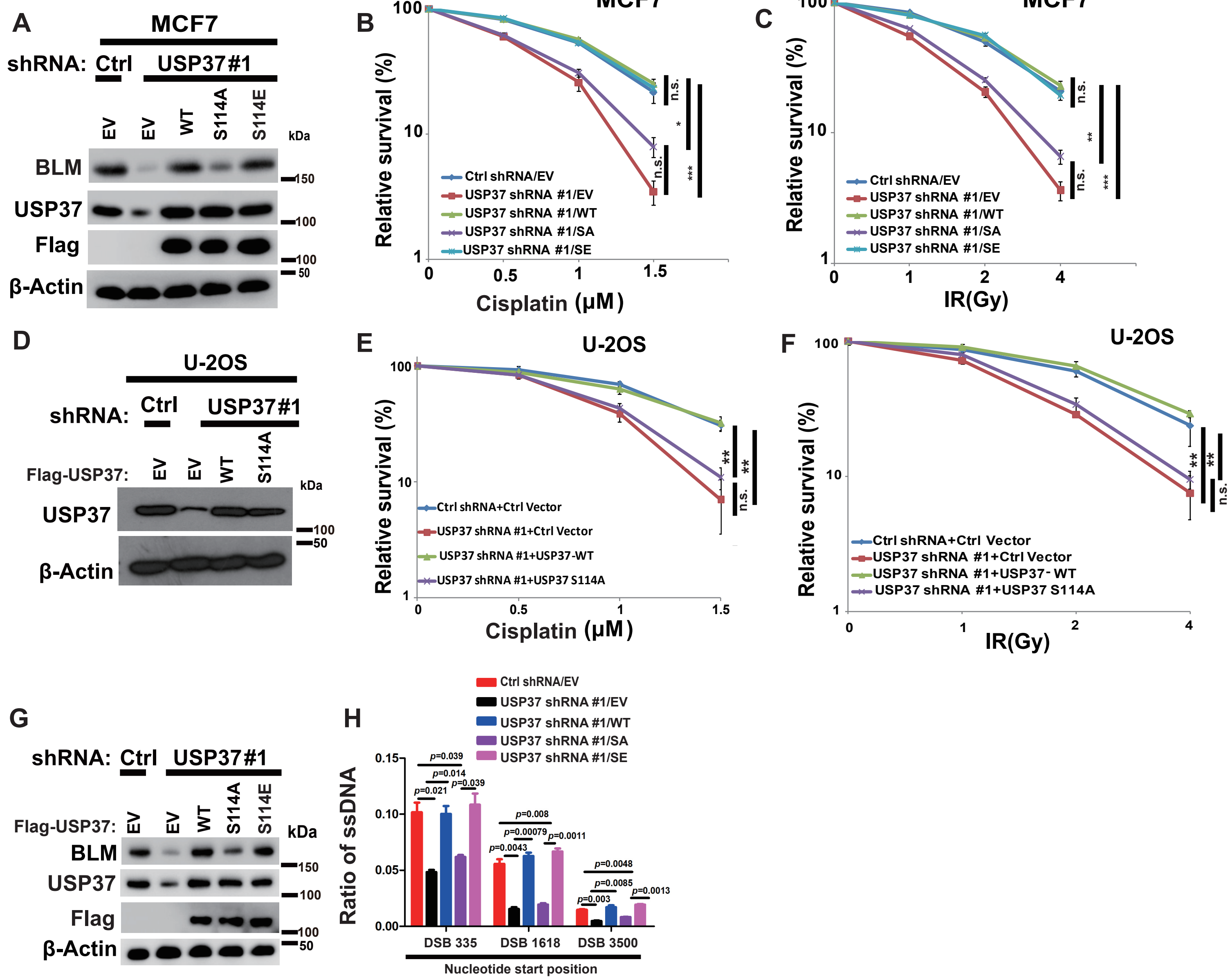

Supplementary Figure S6

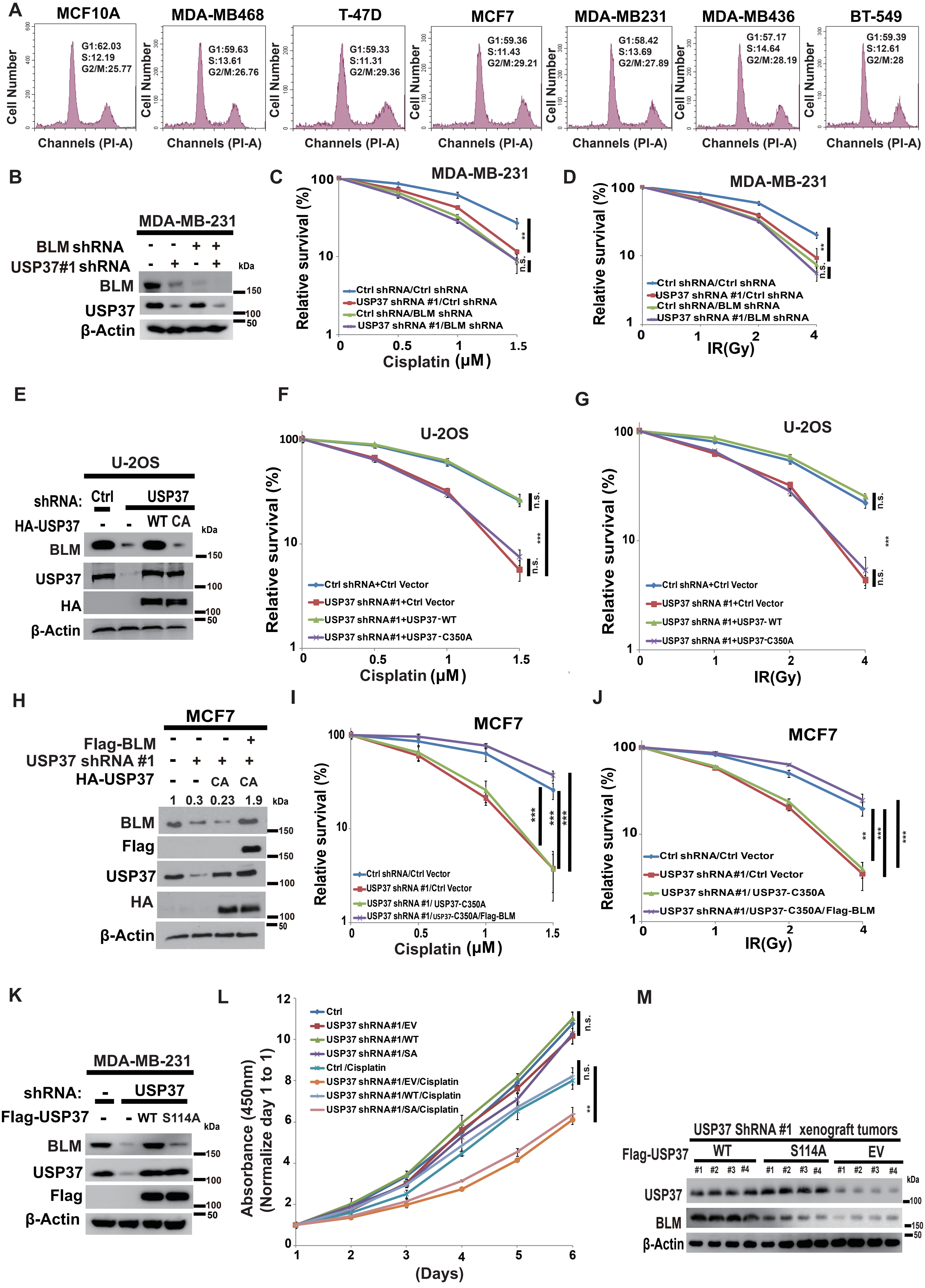

Supplementary Figure S7

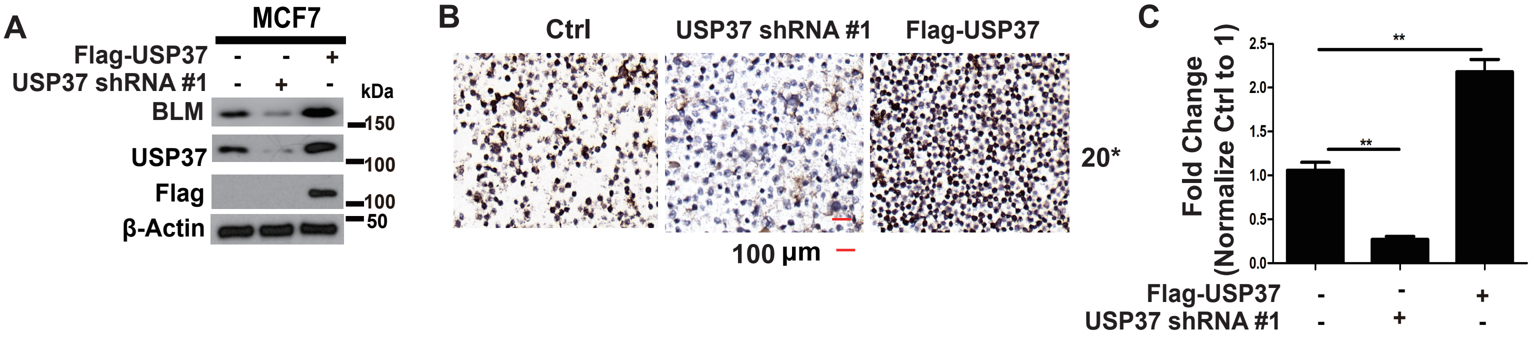

Supplementary Figure S8

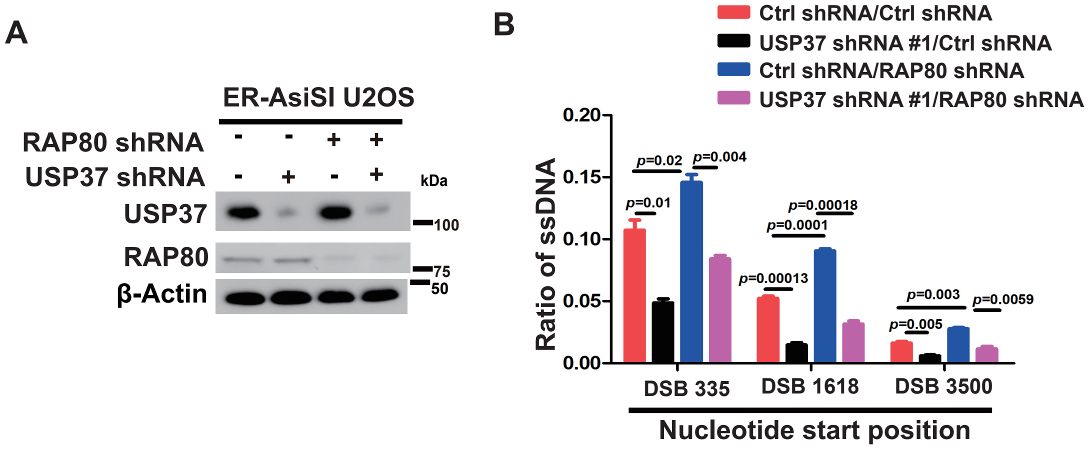

Supplement: gkab842_Supplemental_Files [file gkab842_supplemental_files.zip › Supplementary Figures and Legend.pdf]
