# Supplementary material for: USP37 regulates DNA damage response through stabilizing and deubiquitinating BLM
Source: Nucleic Acids Res. 2021 Oct 4;49(19):11224–40. doi: 10.1093/nar/gkab842 (PMC8565321; doi:10.1093/nar/gkab842)
Supplement: gkab842_Supplemental_Files [file gkab842_supplemental_files.zip › Supplementary table 1 legend.docx]

**Supplementary table 1: The detailed information of the tissue microarray of breast cancer samples.**
