# Supplementary material for: USP37 regulates DNA damage response through stabilizing and deubiquitinating BLM
Source: Nucleic Acids Res. 2021 Oct 4;49(19):11224–40. doi: 10.1093/nar/gkab842 (PMC8565321; doi:10.1093/nar/gkab842)
Supplement: gkab842_Supplemental_Files [file gkab842_supplemental_files.zip › Supplementary table 2.docx]

**Supplementary table 2. Sequence of qPCR primers and probes were used in DNA resection assays.**

| **DSB1-335 bp** | **Primer FW** | **GAATCGGATGTATGCGACTGATC** |
| --- | --- | --- |
|  | **Primer REV** | **TTCCAAAGTTATTCCAACCCGAT** |
|  | **Probe** | **6FAM-CACAGCTTGCCCATCCTTGCAAACC-TAMRA** |
| **DSB1-1618 bp** | **Primer FW** | **TGAGGAGGTGACATTAGAACTCAGA** |
|  | **Primer REV** | **AGGACTCACTTACACGGCCTTT** |
|  | **Probe** | **6FAM-TTGCAAGGCTGCTTCCTTACCATTCAA-TAMRA** |
| **DSB1-3500 bp** | **Primer FW** | **TCCTAGCCAGATAATAATAGCTATACAAACA** |
|  | **Primer REV** | **TGAATAGACAGACAACAGATAAATGAGACA** |
|  | **Probe** | **6FAM-ACCCTGATCAGCCTTTCCATGGGTTAAG-TAMRA** |
