# Supplementary figures and images for: Correction to ‘USP37 regulates DNA damage response through stabilizing and deubiquitinating BLM’
Source: Nucleic Acids Res. 2023 Feb 6;51(5):2496–7. doi: 10.1093/nar/gkad070 (PMC10018356; doi:10.1093/nar/gkad070)

Figure 6

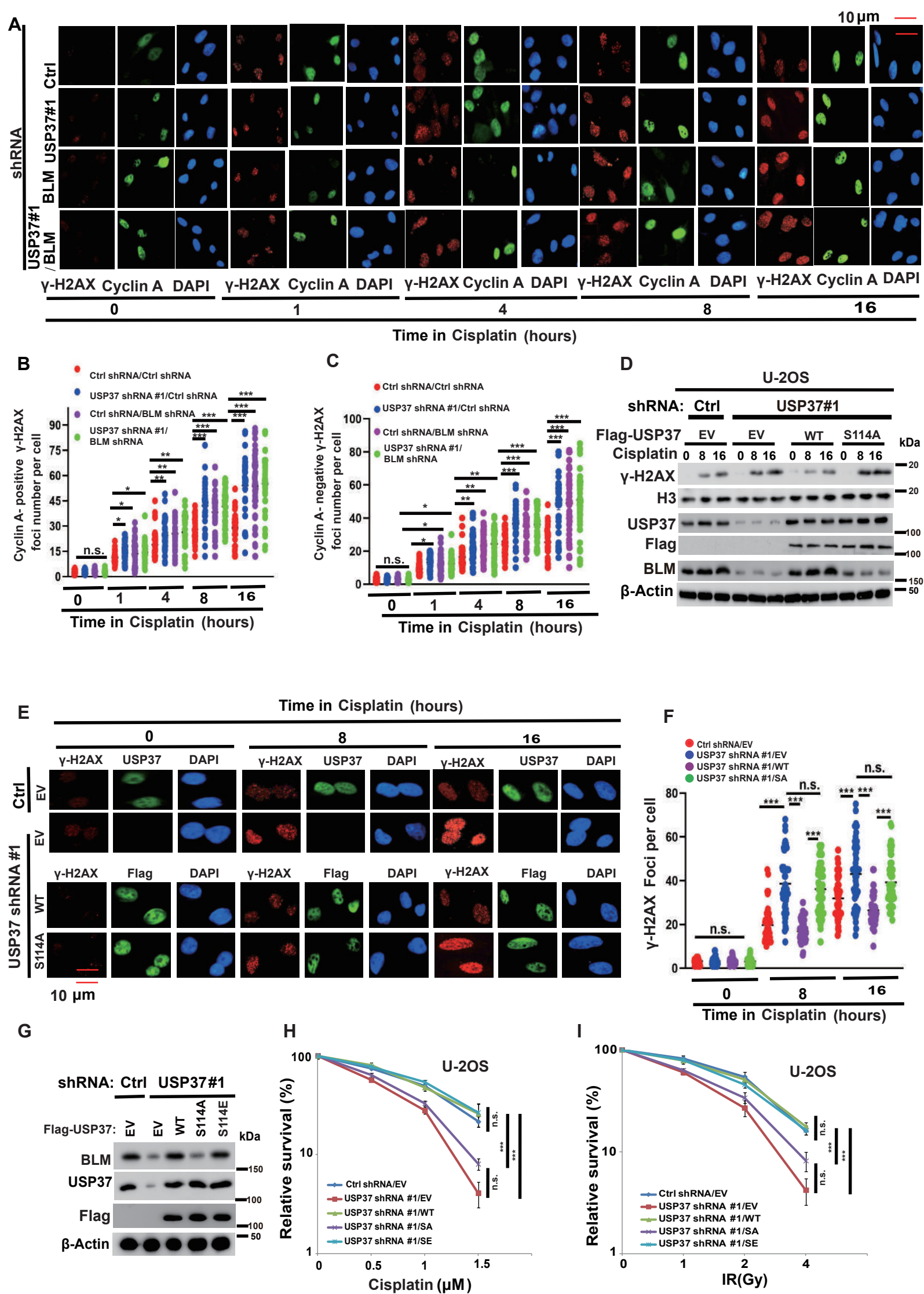

Figure 7

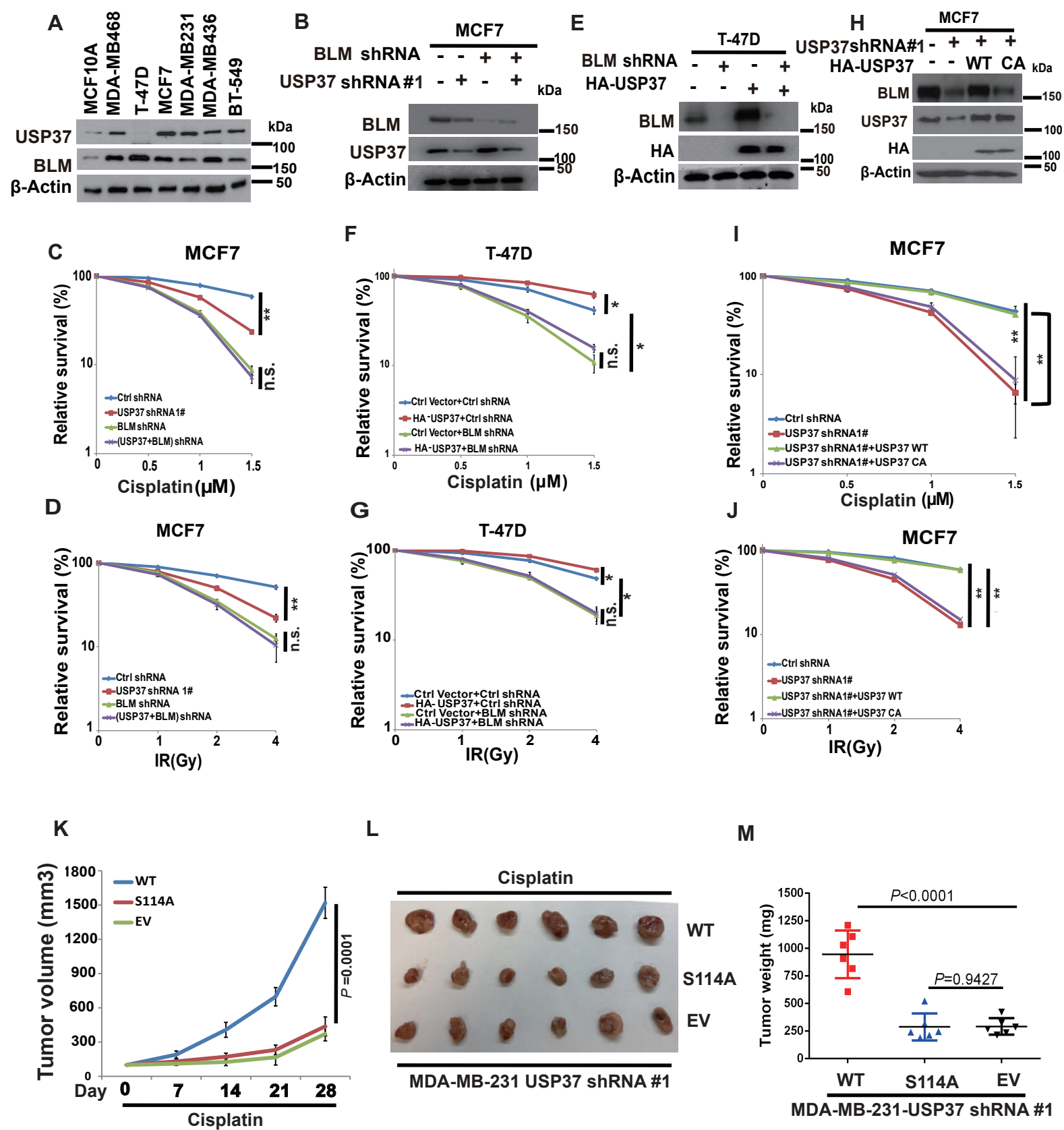

Supplementary Figure S4

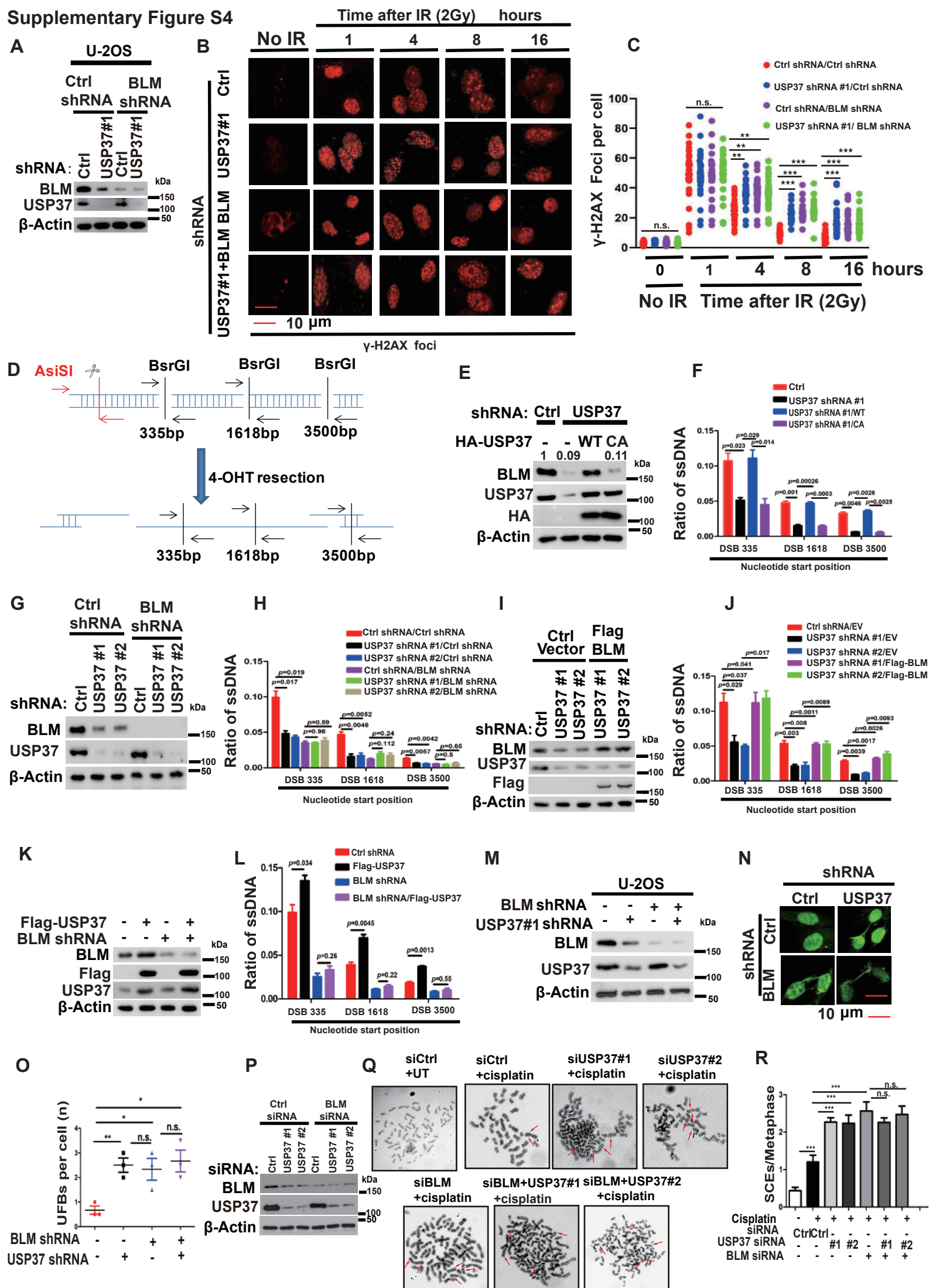

Supplement: gkad070_Supplemental_File [file gkad070_supplemental_file.pdf]
